# Longitudinal studies examining the impact of prenatal and subsequent episodes of maternal depression on offspring antisocial behaviour

**DOI:** 10.1007/s00787-019-01447-w

**Published:** 2019-12-02

**Authors:** Joanne E. Morgan, Sue Channon, Helen Penny, Cerith S. Waters

**Affiliations:** School of Psychology, Cardiff University, Cardiff, Wales CF10 3AT UK

**Keywords:** Maternal depression, Prenatal, Postnatal, Longitudinal, Child antisocial behaviour

## Abstract

Maternal depression is associated with adverse child outcomes including antisocial behaviour (ASB). Prospective longitudinal studies have focused on the timing and cumulative exposure to maternal depression to further delineate the association and mechanisms of effect. The objective of this systematic review was to synthesise and evaluate the findings of longitudinal studies of maternal depression and offspring antisocial behaviour. Three databases were searched (Psychinfo, Web of Science, and Medline). Twenty of 5936 studies met inclusion criteria. Study quality was assessed using the Critical Appraisal Skills Programme criteria [Critical Appraisal Skills Programme (2017) CASP (cohort observation checklist). https://casp-uk.net/wpcontent/uploads/2018/01/CASP-Cohort-Study-Checklist.pdf]. Results of individual studies were highly varied, using diverse analytical approaches and not all studies explored the independent effects of different episodes. Only three studies examined hypothesised mechanisms. Prenatal, postnatal, and later episodes of depression were all predictive of antisocial outcomes. One particular time period of depression exposure did not emerge as more predictive of offspring ASB than another. However, measures of maternal depression after the perinatal period were limited and typically included a one-off assessment of mothers’ depressive symptoms that was concurrent to the assessment of offspring ASB. When cumulative exposure to maternal depression and specific timing effects were measured within the same study it was cumulative exposure that conferred the greatest risk for offspring ASB—particularly when this exposure began during the perinatal period. Findings are discussed in terms of limitations in the literature and highlight the need for future research to examine the biological and environmental mechanisms that underpin associations between maternal depression and offspring antisocial behaviour during different stages of development.

**Electronic supplementary material:**

The online version of this article (10.1007/s00787-019-01447-w) contains supplementary material, which is available to authorized users.

## Introduction

Maternal depression is recurrent and particularly common in the childbearing years; affecting mothers during pregnancy and the postnatal period with a prevalence ranging from 8 to 13% [2, 3]. The importance of maternal mental health and the implications for children’s development has gained increased recognition in recent years. Guidelines in the United Kingdom (UK) have emphasised the importance of screening and improved intervention during the perinatal period which encompasses pregnancy and the first year after birth [4–6]. Interestingly, studies that have examined maternal depression beyond the perinatal period (e.g. occurring during early to late childhood and/or adolescence) show similar or even higher prevalence rates [7–9]. The impact of these recurring or new onset episodes of maternal depression beyond the perinatal period on children’s subsequent outcomes also requires consideration [8, 9]. While it is recognised that maternal depression and anxiety are moderately correlated during the perinatal period and that both are associated with adverse offspring outcomes [4], the focus of this review is on the independent effects of maternal depression.

Maternal depression has been associated with a range of adverse child outcomes including offspring antisocial behaviour. Antisocial behaviour (ASB) in children is a serious public health concern; it incurs significant costs for society and for the individuals involved who are at increased risk for substance use, chronic health problems, and early mortality [10]. By examining early predictors of ASB it is hoped that more targeted and early intervention strategies can be applied. Maternal depression in both the prenatal and postnatal period is associated with increased offspring antisocial behaviour [11–15]; although debate exists as to whether effects are unique to these time periods or represent exposure to mother’s lifelong episodic illness [16]. Indeed, research on maternal depression and child psychopathology has turned its attention away from whether relationships exist to attempt to understand the specifics of the complex associations, including an examination of the potential mechanisms of effect [17]. The timing of exposure to maternal depression is of interest as this provides an avenue through which to explore potential developmental mechanisms with implications for appropriate intervention for both mother and child [17].

Exposure to maternal depression at different time points through a child’s development is thought to exert effects on offspring antisocial behaviour via different biological, environmental, and epigenetic mechanisms. This literature is complex and expanding with a focus in the prenatal period on testing the *fetal programming hypothesis* [18]. Drawing on the animal literature that has identified mechanisms underlying the association between prenatal stress and offspring cognition and behaviour including the role of the HPA axis, the placenta, and epigenetics [19, 20], studies in humans have focused on the prenatal exposure to stress and anxiety as well as depression. While studies in humans are not as advanced or well replicated as the animal literature, downregulation of placental 11-beta-Hydroxysteroid Dehydrogenase Type 2 (the enzyme which metabolises cortisol) in prenatally anxious mothers is thought to enable increased cortisol to pass from mother to foetus affecting the offspring’s developing brain [21]. In line with the fetal programming hypothesis, Monk and colleagues [22] have shown that increased prenatal stress is associated with DNA methylation of glucocorticoid related genes which in turn is associated with reductions in foetal coupling—a key foetal outcome predictive of offspring neurobehavioural development [22]. Individual differences in DNA methylation assessed via a number of different paradigms has been prospectively associated with the emergence of offspring ASB [23–25].

Replicating findings from the animal literature that has highlighted epigenetic processes operating across the pre-and-postnatal environments [26–28], the frequency of maternal stroking has been shown to moderate the effect of prenatal depression on infant behavioural and physiological outcomes [29]. Other studies have shown a dose response relationship between exposure to prenatal risk factors (including maternal depression), fearless temperament in early childhood and the development of offspring conduct problems and callous unemotional traits in adolescence [30]. As the human literature develops, complex biological and epigenetic mechanisms are discovered with sex-specific and ethnicity-specific effects reported [31, 32]. Whilst it is recognised that the association between maternal depression and adverse offspring outcomes at least in part reflects shared genetic risk, genetically informative designs have shown that both genetic and environmental mechanisms contribute to the elevated risk of antisocial behaviour among the offspring of depressed parents [14, 33].

The quality of the care-giving environment and the parent–child relationship has long been highlighted as key mechanisms underpinning the association between pre- and postnatal depression and adverse offspring outcomes [34–36]. Postnatal depression is associated with decreased maternal sensitivity (e.g. increased intrusiveness, reduced responsiveness, disengagement or negativity) and increased offspring attachment insecurity [34, 37–39]. In the context of postnatal depression, reduced maternal sensitivity and poor offspring emotion regulation capacities first evident in infancy have been linked to elevated rates of behavioural problems at age five years [40]. Other studies identify *maternal programming effects* where depression during pregnancy is hypothesised to impair the psychological and cognitive processes that prepare women to respond appropriately towards their infants [41, 42]. Prenatal depression is associated with decreased maternal responsiveness independently of postnatal depression [41], and maternal sensitivity has been shown to mediate the relationship between prenatal depression and offspring behavioural problems [43]. Other environmental stressors known to correlate with maternal depression have been highlighted as potential mechanisms including inter-parental conflict and domestic violence [44, 45]. For example, inter-parental conflict partially mediates the relationship between postnatal depression and offspring behavioural problems [44]. Mechanisms that underpin the association between depression after the perinatal period have been implicated including social learning processes following exposure to negative maternal cognitions, affect and behaviour, inadequate parenting including physical punishment, and the impact of stress on family communication processes including increased inter-parental conflict [36, 46].

Longitudinal studies that examine maternal depression at multiple time points and begin during the pregnancy period are essential for examining the independent and cumulative effects of particular episodes and indeed the relative importance of exposure during specific developmental time periods. Longitudinal studies have found that prenatal and postnatal depression can have different effects on child and adolescent outcomes [47]. Other studies have demonstrated that perinatal (prenatal and postnatal) depression predicts later child problems after controlling for later episodes of maternal depression occurring during early, middle or late childhood [48]; while others have found perinatal depression to be less important and stronger associations have been documented for maternal depression in late childhood and adolescence [49]. This emerging evidence on the complex relationships between exposure to prenatal, postnatal, and later episodes of maternal depression (e.g. episodes occurring during early to late childhood or adolescence) and adverse offspring outcomes lacks cohesion; while reviews exist on the relationship between maternal depression and child outcomes (e.g. [13, 15]), there has been no systematic review of these emerging longitudinal studies that begin during the prenatal period.

Maternal depression has been associated with a range of adverse offspring outcomes with empirical studies and reviews often exploring and comparing a diverse set of outcomes. The present review seeks to focus in and examine the contribution of maternal depression to offspring antisocial behaviour in particular. Antisocial behaviour is a heterogeneous construct and the extent to which specific domains of behaviour are implicated has not been given sufficient attention in these broader examinations of child outcomes. Indeed, ASB has been operationalised in diverse ways in the literature [50, 51]. Within empirical studies there is a tendency to use terminology related to the measures that are utilised. Widely used questionnaires such as the Child Behaviour Checklist (CBCL) and the Strengths and Difficulties Questionnaire (SDQ) refer to externalising behaviour or conduct problems. Whereas another prominent approach is the examination of clinical diagnoses including Oppositional Defiant Disorder or Conduct Disorder assessed via standardised clinical interviews such as the Development and Wellbeing Assessment (DAWBA) or the Child and Adolescent Psychiatric Assessment (CAPA). Legal definitions such as delinquency are also relevant in older children. Also worthy of note here is the significant body of research that has focused on the presence of Callous-Unemotional traits (e.g. lack of guilt, remorse, shallow affect) as a way to identify a more severe and persistent subgroup of antisocial children [52, 53]. The aim of this systematic review is to be all encompassing through using the term antisocial behaviour in the widest sense to capture evidence that incorporates any of these approaches to the measurement of offspring antisocial behaviour.

More specifically, the aims of this systematic review were (i) to identify studies that have assessed maternal depression during the prenatal period and at least once following birth and examined the effect on child antisocial behaviour outcomes, (ii) to evaluate whether these studies examine the independent and/or cumulative effects of prenatal, postnatal and subsequent episodes on child antisocial behaviour and describe and synthesise the findings accordingly, (iii) to examine whether these studies have examined biological or environmental mechanisms that underpin any associations between maternal depression and offspring antisocial behaviour, (iv) describe the quality of the available evidence, consider study limitations and identify gaps in the existing evidence base, and (v) describe the implications and make clinical, research and policy recommendations.

## Method

The systematic review was conducted following recommendations outlined in the Preferred Reporting Items for Systematic Reviews and Meta-Analysis Protocols [PRISMA-P] statement [54]. The PRISMA guidelines include a flow diagram to guide inclusion and exclusion of research articles and provide a 27-item checklist outlining appropriate standards for conducting a review [54]. The methods of analysis and inclusion criteria were specified in advance and documented in a protocol.

### Inclusion and exclusion criteria

Studies were included if: (i) the initial exposure was prenatal maternal depression with subsequent exposure to at least one episode of maternal depression following birth; (ii) the outcome was a measure of child antisocial behaviour that was assessed at any age from birth onwards; (iii) the study was published in English; (iv) it was a longitudinal prospective quantitative study; (v) the study was a primary study that was published between January 1900 and December 2017. Studies were excluded from this review if: (i) maternal depression was part of a composite variable and could not be distinguished from anxiety measures or general stress; (ii) the exposure was a pharmacological intervention for maternal depression; (iii) it was an animal study; (iv) they combined mother and father data into parent’s depression. In addition, grey literature articles were excluded, as this literature is not peer reviewed it may introduce bias to the findings [55]. The comorbidity of perinatal anxiety and depression is acknowledged and important to highlight. When a study assessed both perinatal depression and anxiety we focus on the findings that are unique to perinatal depression, and document whether the impact of perinatal anxiety was accounted for in statistical analyses in the covariates column of Table 2.

### Definitions

#### Maternal depression

Studies of depression that included both self-report questionnaires and diagnostic interviews were included. The prenatal period was defined as any time during pregnancy up until the birth of the child. For the *postnatal period* the review adopts the widely used timeframe from birth to 12 months’ post birth [8, 56]; episodes of depression outside of this time were referred to as later exposure and the age of the child when maternal depression was assessed is reported. The developmental time periods of early (1–4 years), middle (5–8 years) or late childhood (9–12 years), and adolescence (13–18 years) are used to summarise when the child was exposed to maternal depression. *Concurrent depression* refers to the assessment of depression at the same time as the assessment of the child outcome. *Cumulative exposure* refers broadly to exposure to maternal depression at more than one-time point and encompasses reference to terms such as recurrence, chronicity and persistence. These terms are defined and operationalised in diverse ways in the literature and will be referred to and defined if and when they occur.

#### Child antisocial behaviour

As noted child antisocial behaviour (ASB) is heterogeneous and it was important to encompass as many different operationalisations of this construct as possible as well as allow for the capture of early manifestations (e.g. angry moods, aggression). There is substantial comorbidity among childhood psychological problems [13, 57] and many studies used symptom checklists that result in scores that are summarised by two broad constructs, behavioural problems (*externalising*) and emotional problems (*internalising*). The main focus of the review is on the externalising element, so studies were included only if a measure of behavioural problems was incorporated. Unless specific domains of the ASB construct were assessed (e.g. violence or diagnoses of disruptive behaviour disorders) then the term *behavioural problems* is used in Table 3. Studies that used standardised symptom checklists (e.g. CBCL or SDQ) and reported on composite scores that contained a heavily behavioural problems component were included. Studies that used measures that assessed specific dimensions of the ASB construct (e.g. symptom ratings of conduct problems, violence and aggression, criminal activity/arrest history, psychiatric diagnoses of conduct disorder/oppositional defiant disorder) were included.

### Information sources and search strategy

Three electronic databases relevant to the research aims were searched; Medline, Psychinfo (OVID), Web of Science. The search encompassed the period from January 1st 1900 to December 31st 2017. The search terms utilised were a combination of database-specific index terms (e.g. postnatal depression, child development) and individual terms located in the title, abstract or key words. These terms were combined with the necessary Boolean (AND; OR) proximity (ADJ3 or NEAR/3) truncation (*) and phrasing (“ ”) operators.

More specifically, the maternal depression terms used included (“maternal depress*” OR “antenatal depress*” OR “postnatal depress*” OR “perinatal depress*” OR “postnatal depress*” OR “postpartum depress*” OR “antepartum depress*”) combined with the child terms (child* OR infant* OR adolescent* OR teen* OR toddler* OR baby OR babies OR youth OR offspring) ADJ3 (behaviour* OR behavior* OR development OR externalis* OR conduct problem* OR conduct disorder* OR psychopathology OR callous-unemotional OR maladjust* OR aggress* OR violent* OR anger* OR disruptive behaviour disorder* OR oppositional defiant disorder* OR emotion* OR cognit* OR youth offend* or juvenile delinquency OR antisocial.* A copy of the exact search strategy with all terms used in the Psychinfo database search is available from the first author.

In addition, the ancestor method was used in which any relevant references listed in empirical articles or review articles were retrieved. It is likely that other relevant studies exist and were not identified, however, it is argued that the scope of the search was suitable for identifying a representative sample of the relevant papers. Furthermore, PROSPERO (International prospective register of systematic reviews) was examined to assess if any similar reviews were currently registered to be undertaken; personal communication with two of the principal investigators of two proposed studies confirmed that the current review was suitably distinct. The papers identified in each of the databases and other sources were imported and combined within the Mendeley Reference Management System which automatically removed the majority of duplicates; the remainder of the duplicates were removed manually. The titles and/or abstracts of each journal article was reviewed by one individual based on the a priori inclusion and exclusion criteria; those meeting the inclusion criteria were selected for full text evaluation.

### Quality appraisal, data extraction, analysis, ethics

The selected full text articles were appraised based on study design, potential for selection bias, confounding variables, attrition, follow up, blinding and measures of exposures and outcomes. The Critical Appraisal Skills Programme (CASP) guidance [1] recommended for the appraisal of observational cohort research was used to systematically review the quality of the studies. This particular framework provides a tool for appraising cohort studies across 12 domains (and includes a series of questions in relation to each domain that can be responded with a yes/no/cannot tell). To compare studies more readily, for each domain, a score of 2 was given for yes ‘criteria met’, 1 for ‘partially met’, and 0 for ‘not met’ and the scores summed to give a maximum quality score of 24. A qualitative descriptor was then provided based on the score, number of criteria met and the risk of bias (following the Scottish Intercollegiate Guidelines Network framework [58]). The following descriptors were used: “High Quality” = majority of criteria met; little or no risk of bias; results unlikely to be changed by further research (a score of 18–24), “Acceptable” = most criteria met; some flaws in the study with associated bias (a score of 12–17), and “Low quality” = either most criteria not met or significant flaws relating to key aspects of study design (below a score of 12).

The quality of the full text articles was assessed by two independent reviewers with experience in critical appraisal. Disagreement was resolved by coming to a consensus. Following accepted procedures [59], the reliability between the raters was assessed based on agreement of the qualitative descriptors (low; acceptable; high) for 10 studies. Percentage agreement was 80% (Kappa = 0.6). Please see Table 1 for a summary of the quality ratings for each study. A descriptive (qualitative) analysis of the findings was planned and studies were also reviewed for the potential to conduct a meta-analytical review. Data extraction from the 20 papers was conducted by two independent reviewers to ensure accuracy. This review was exempt from ethics review and approval.

**Table 1:** CASP quality ratings (*N* = 20)

References	Barker et al. [64]	Brennan et al. [49]	Edwards and Hans [43]	Eichler et al. [65]	Gjerde et al. [61]	Hannington et al. [44]	Hay et al. [51]	Hay et al. [48]	Hay et al. [47]	Korhonen et al. [70]	Korhonen et al. [69]	Lahti et al. [66]	Leis et al. [71]	Luoma et al. [67]	O’connor et al. [62]	O’Donnell et al. [72]	Raskin et al. [60]	Soe et al. [63]	van de Waerden et al. [68]	Woolhouse et al. [9]
1. Did the study address a clearly focused issue? In terms of…^a^	2	2	2	2	2	2	2	2	2	2	2	2	2	2	2	2	2	2	2	2
Is the population studied clear?^b^	Y	Y	Y	Y	Y	Y	Y	Y	Y	Y	Y	Y	Y	Y	Y	Y	Y	Y	Y	Y
Are the factors studied clear?^b^	Y	Y	Y	Y	Y	Y	Y	Y	Y	Y	Y	Y	Y	Y	Y	Y	Y	Y	Y	Y
Are the outcomes clear?^b^	Y	Y	Y	Y	Y	Y	Y	Y	Y	Y	Y	Y	Y	Y	Y	Y	Y	Y	Y	Y
2. Was the cohort recruited in an acceptable way? (selection bias)^a^	1	0	0	1	0	1	1	1	1	1	1	1	1	1	1	1	0	0	1	1
Was the cohort representative of a defined population?^b^	P	P	N	N	P	P	Y	Y	Y	P	P	P	P	P	P	Y	N	P	P	P
Was everybody included who should have been included?^b^	P	N	P	P	N	P	P	P	P	P	P	P	P	P	P	P	N	N	N	N
3. Was the exposure accurately measured to minimise bias?^a^	1	0	1	1	1	1	1	1	1	1	1	1	1	1	1	1	1	1	1	1
Did they use objective measurements?^b^	N	N	N	N	N	N	P	P	P	P	P	P	P	P	N	P	N	N	N	N
Do the measurements truly reflect what you want them to?^b^	Y	P	Y	Y	Y	Y	Y	Y	Y	Y	Y	Y	Y	Y	Y	Y	Y	Y	Y	Y
4. Was the outcome accurately measured to minimise bias?^a^	1	1	1	0	1	1	1	2	2	1	1	1	1	1	1	1	1	1	1	1
Did they use objective measurements?^b^	P	P	P	P	P	P	Y	Y	Y	P	P	P	P	P	N	P	N	N	N	N
Do they measures truly reflect what you want them to^b^	Y	P	Y	N	Y	Y	Y	Y	Y	Y	Y	Y	Y	Y	Y	Y	P	Y	Y	Y
Was there blinding to exposure?^b^	N	N	N	N	N	N	P	Y	Y	P	P	P	P	P	?	P	N	N	P	P
5. Have the authors identified all important confounding factors?^a^	2	2	1	0	2	2	2	2	2	0	0	2	2	1	1	2	1	2	2	2
Have they taken into account the confounding factors in design/analysis?^b^	Y	Y	P	P	Y	Y	Y	Y	Y	N	N	Y	Y	P	P	Y	P	Y	Y	Y
6. Was the follow up of subjects complete enough?^a^	2	2	2	2	2	2	2	2	2	2	2	2	2	2	2	2	2	2	2	2
Was the follow up of subjects long enough?^b^	Y	Y	Y	Y	Y	Y	Y	Y	Y	Y	Y	Y	Y	Y	Y	Y	Y	Y	Y	Y
7. What are the results of the study? Are they reported clearly?^a^	2	2	1	1	2	2	2	2	2	1	2	2	2	2	1	1	1	2	2	2
Have they reported the rate or the proportion between exposed^b^	Y	Y	Y	Y	Y	Y	Y	Y	Y	Y	Y	Y	Y	Y	Y	Y	N	Y	Y	Y
8. How precise are the results?	1	2	1	1	2	2	1	1	2	2	2	2	1	2	2	1	1	1	2	2
Were confidence intervals given?^b^	N	Y	N	Y	Y	Y	N	N	Y	Y	Y	Y	N	Y	Y	N	N	N	Y	Y
9. Do you believe the results?^a^	2	1	2	1	2	2	2	2	2	1	1	2	2	2	2	2	1	1	2	2
Could it be due to bias, chance or confounding?^b^	N	P	N	P	N	N	N	N	N	P	P	N	N	N	P	N	N	N	N	N
Are the design/methods of this study flawed to make the results unreliable?^b^	N	N	N	N	N	N	N	N	N	N	N	N	N	N	N	N	N	N	N	N
10. Can the results be applied to the local population?^a^	2	0	0	0	1	2	2	1	1	1	1	1	1	1	1	1	0	0	1	1
Was the cohort the appropriate method?^b^	Y	Y	Y	Y	Y	Y	Y	Y	Y	Y	Y	Y	Y	Y	Y	Y	Y	Y	Y	Y
11. Do the results of this study fit with other available evidence?^a^	2	1	2	2	2	2	2	2	2	2	2	2	2	2	2	2	1	0	2	1
12. Implications of this study for practice? Are they justified?^a^	1	1	1	1	1	1	1	1	1	1	1	1	1	1	1	1	1	1	1	1
Overall Score^a^	19	14	14	12	18	20	19	19	20	15	16	19	18	18	17	17	12	13	19	18
Qualitative Descriptor **(**0 unacceptable; + acceptable; ++ High Quality**)**^a^	++	+	+	+	++	++	++	++	++	+	+	++	++	++	+	+	+	+	++	++

^a^The main questions are the 1–12 (score 0 = criterion not met, 1 = mixed evidence, 2 = criterion met)

^b^The questions prompt to the main questions to aid scoring (Y = yes; N = no; P = partial

## Results

### Overview of studies

**Fig. 1:**
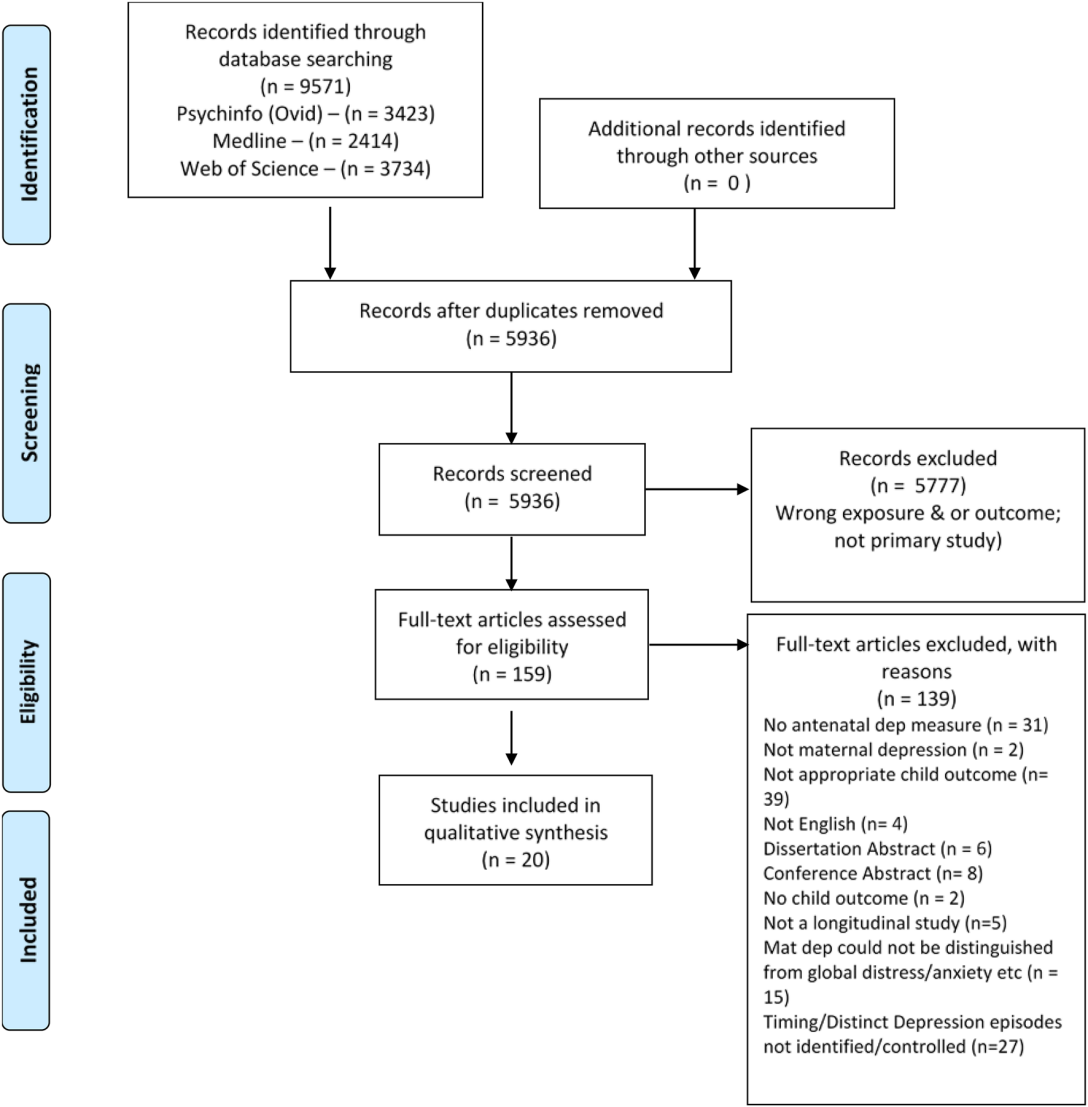
PRISMA flow chart

5936 studies were identified by the search terms after removal of duplicates. These studies were systematically screened according to the exclusion and inclusion criteria. The main reasons for exclusion included wrong exposure (including distress/anxiety rather than depression being measured, not assessing depression on more than one occasion, no prenatal measure of depression, etc.), wrong outcome (not a measure of ASB as defined above), and also design and/or analysis not focussed on the examination of timing of depressive episodes (e.g. combining episodes into depressed or not, depression used as a mediator/moderator for other off topic analyses). This process resulted in 159 articles downloaded for additional screening with a further 139 excluded, thus resulting in 20 studies for inclusion in the systematic review. Please see Fig. 1 for the PRISMA flow diagram and details of this screening process. The list of excluded articles is available from the first author.

The key study characteristics extracted from the 20 studies are summarised in Table 2. The study publication dates ranged from 2000 to 2017; with the majority (*n* = 13 studies) being published in the last five years. Of the 20 included studies, eight developed countries were represented (United Kingdom, Australia, United States, Finland, Germany, France, Norway, and Singapore). The studies identified examined 10 different cohorts; five papers were based on the Avon Longitudinal Study of Parents and Children (ALSPAC), a large cohort study in Bristol, England; while three came from the South London Child Development Study (SLCDS); and three from the Finland Tampere longitudinal study.

**Table 2:** Study characteristics (*N* = 20)

Study	Location and study	Participants^a^	Child age	Measures (mother/child/timing of administration/mechanisms)	Covariates	Data analysis	Tests of mediators/moderators	Tests of gender
Barker et al. [64]	England ALSPAC	3298 mothers 24.3 years during pregnancy 8.3% low SES 3.7% single	7–8 years	Prenatal depression: Mother report, EPDS, 32 weeks Postnatal depression: None Later depression: Mother report, EPDS, 1.5 years Child outcome: Parent and teacher report, DAWBA, Externalising disorders (CD; ODD; ADHD) Mechanisms: N/A	SES, marital status, teenage mother, substance use, criminal behaviour, cigarette smoking, prenatal and postnatal anxiety symptoms	Single path analytic model	No	Yes
Brennan et al. [49]	Australia	4953 mothers 25.4 years at birth 92% Caucasian Working-lower middle class sample	5 years	Prenatal depression: Mother report, DSSI (trimester not specified) Postnatal depression: Mother report, DSSI, 3–4 days after birth and 6 months postpartum Later depression: Mother report, DSSI, 5 years Child outcome: Mother report, CBCL short form, total problems (aggression, oppositional behaviour, hyperactivity, anxiety, withdrawal, and depression) Mechanisms: N/A	Gender and birth order of child; maternal age and education; family income; number of changes in marital status. Did not assess prenatal/postnatal anxiety	Multiple regression ANCOVA	No	Yes
Edwards and Hans [43]	United States	196 mothers 18.3 years at birth All low income African American 69% in a relationship with biological father	2 years	Prenatal depression: Mother’s report, CES-D, pregnancy (trimester not specified) Postnatal depression: Not measured/reported Later depression: Mother’s report, CES-D, 24 months Child outcome: Mother report on BITSEA–Toddler behavior problems total score (externalising, dysregulation, atypical behaviours, and maladaptive behaviours) Mechanisms: Maternal sensitivity coded using the Parent–Child Observation Guide (PCOG) assessed at 24 months	Neonatal problems (pre-term birth, low birth weight, Apgar score), neonatal special care admission; maternal age, smoking and verbal ability; parental relationship quality. Did not assess prenatal/postnatal anxiety	Path analysis	Yes	Yes
Eichler et al. [65]	Germany Franconian Cognition and Emotion Studies	204 mothers 32.8 years at birth 10% ethnic minority 87% living with biological father Average family income 3000-4000 Euros	6-9 years	Prenatal depression: Mother report, EPDS, third trimester Postnatal depression: Not measured/reported Later depression: Mother report, EPDS, 6-9 years Child: Mother report, Antisocial behaviour symptoms questionnaire, items of the diagnostic system for psychiatric disorders ICD-10	Family status, gestational age, APGAR score, offspring gender. Did not assess prenatal/postnatal anxiety	ANCOVA	No	Yes
Gjerde et al. [61]	Norway Norwegian Mother and Child Cohort Study (MoBa)	11,599 mothers Demographics not reported	1.5, 3, and 5 years	Prenatal depression: Mother report on depression, short form of the SCL, 17 weeks, 30 weeks Postnatal depression: Mother report on depression, short form of the SCL, 6 months Later depression: Mother report on depression, short form of the SCL, 1.5, 3 and 5 years Child: Mother report on CBCL externalising scale	Child age, gender, maternal parity, education, familial confounding via a sibling comparison. Did not assess prenatal/postnatal anxiety	Latent growth curve analysis	Yes	No
Hanington et al. [44]	England ALSPAC	14,541 mothers 97.4% white 50.6% skilled workers	3 years	Prenatal depression: Mother completed the EPDS 2nd trimester Postnatal depression: Mother completed the EPDS, 8 months Later depression: None Child: Mother report on Rutter Revised pre-school scales (conduct problems) 42 months	Marital conflict, maternal education, paternal depression. Did not assess prenatal/postnatal anxiety	Logistic regression Mediation analysis	Yes	No
Hay et al. [51]	England SCLDS	122 mothers 25.8 years at birth 72% Caucasian 89% working class	11 years	Prenatal depression: Trained doctors conducted the CIS at 14 weeks and 36 weeks Postnatal depression: CIS at 3 months, 1 year Later depression: Trained research assistants administered SADS at 4 years, 11 years Child outcome: Mother, teachers and children reported on violent symptoms using SDQ, research assistants conducted the CAPA with primary care-giver and children at aged 11 years	Social class, financial problems, family structure, parental criminality, child sex. Did not assess prenatal/postnatal anxiety	Structural equation modelling	No	Yes
Hay et al. [48]	England SCLDS	120 mothers 26.7 years at birth 65% married at birth 88% working class 72% Caucasian	16 years	Prenatal depression: Two doctors interviewed mothers using the CIS to generate ICD-9 diagnoses of depression at 14 and 36 weeks Postnatal depression: Two doctors interviewed mothers using the CIS at 3 and 12 months Later depression: Trained researchers administered the SADS at 4, 11 and 16 years Child outcome: Primary care-giver and children interviewed separately using the CAPA. Arrest history. 16 years	Social class, maternal education, cultural background, mother’s age at birth, marital status, two-parent family structure; mother’s intellectual ability; prenatal anxiety symptoms, smoking and drinking; mother’s antisocial behaviour	Logistic regression Markov chain analysis	No	Yes
Hay et al. [47]	England SLCDS	121 mothers 26.2 years 64% married; 88% working class 72% Caucasian	11 and 16 years	Prenatal depression: GPs interviewed mothers, using the CIS, 14-20 weeks, 36 weeks Postnatal depression: GPs interviewed mothers using CIS, 3 months, 12 months Later depression: Mother’s interviewed using the SADS at 4, 11 and 16 years Child outcome: Parents and children interviewed separately using the CAPA, 11 and 16 years	Prenatal anxiety, smoking and alcohol, breastfeeding; mother’s intellectual ability; mother’s symptoms of juvenile conduct disorder	Logistic and linear regression	No	Yes
Korhonen et al. [70]	Tampere, Finland	191 mothers 44 years at last assessment 82% married; 59% upper class	16–17 years	Prenatal depression: Mother report, EPDS, last trimester Postnatal depression: Mother report, EPDS, 2 months Later depression: Mother report, EPDS, 16–17 years old Child outcome: Mother reported, CBCL; Adolescents completed YSR, 16–17 years	Mother’s education; marital status; number of biological children; gender; maternal age. Did not assess prenatal/postnatal anxiety	Linear and logistic regression	No	Yes
Korhonen et al. [69]	Tampere, Finland	191 mothers 44 years at last assessment 82% married; 59% upper social status	16–17 years	Prenatal depression: Mother report, EPDS, 3^rd^ trimester Postnatal depression: Mother report, EPDS, 1 week, 2 and 6 months Later depression: Mother report, EPDS, 4–5, 8–9 and 16–17 years old Child outcome: Mother reported, CBCL; Adolescents completed the YSR, 16-17 years	Analyses of the impact of covariates not reported. Did not assess prenatal/postnatal anxiety	Linear regression and trajectory analyses	No	No
Lahti et al. [66]	Finland Prediction of Preeclampsia (PREDO)	2296 mothers 31.9 years	1.9–5.9 years	Prenatal depression; Mother report on the CES-Depression Scale, biweekly up to 14 times throughout pregnancy beginning 12 weeks Postnatal depression: Not measured/reported Later depression: Mother report on the BDI (1.9–5.9 years) Child outcome: Mother report on the CBCL (1.9–5.9y ears)	Maternal age, parity, education level, family structure. Prenatal smoking, psychotropic drug exposure, chronic hypertension, type 1 diabetes. Child gender, gestational length, birthweight, family structure. Did not assess prenatal/postnatal anxiety	Linear/Tobit regression, mediation analyses	No	No
Leis et al. [71]	England ALSPAC	2891 mothers 29.1 years at birth 98.9% white 83.5% married 42.5% skilled non manual	11 years	Prenatal depression: Mother report, EPDS at 8 weeks, 32 weeks Postnatal depression: Mother report, EPDS, 21 Later depression: Mother report, EPDS 33, 61, 73 months, 11 years Child outcome: Mother and teacher report, SDQ, 11 years	Maternal anxiety symptoms; sociodemographic variables including marital status; maternal age at birth; child birthweight; child gender; maternal education achievement; cigarette smoking	Linear regression	No	No
Luoma et al. [67]	Finland	270 mothers 37.4 years at last assessment	8–9 years	Prenatal depression: Mother report, EPDS 3rd trimester Postnatal depression: Mother report using EPDS, 1 week, 2 months, 6 months postpartum Later depression: Mother report using EPDS, 8–9 years Child outcome: Mother and teacher report, CBCL, TRF 8–9 years	Mother’s education, marital status, age, family SES, number of children in the family, child gender. Did not assess prenatal/postnatal anxiety	Logistic regression	No	No
O’Connor et al. [62]	England ALSPAC	7442 mothers 28 years in pregnancy 45% first time mothers	4 years	Prenatal depression: Mother report, EPDS, 18 weeks, 32 weeks Postnatal depression: Mother report, EPDS, 8 weeks, 8 months Later depression: None Child outcome: Parent report, SDQ (total scale), 47 months	Maternal education, gestational age, smoking, child gender and prenatal anxiety	Logistic and ordinary least regression	No	Yes
O’Donnell et al. [72]	England ALSPAC	7944 mothers 28.22 years at birth	4, 7, 9, 11 and 13 years	Prenatal depression: Mother report, EPDS, 18 weeks, 32 weeks Postnatal depression: Mother report, EPDS, 8 weeks, 33 months Later depression: Mother report, EPDS, 33 months Child outcome: Mother report, SDQ, 4 years, 7 years, 9 years, 11.5 years, 13 years	Prenatal anxiety. Paternal anxiety and depression. Maternal age, education, household crowding, positive parenting behaviours at age 2. Prenatal smoking; alcohol/substance use. Birth weight, gestational age	Trajectory analysis	No	Yes
Raskin et al. [60]	United States	400 mothers 18.5 years at assessment 35% White	2 years	Prenatal depression: Mother report, CES-D, either 1st (14%), 2nd (49%) or 3rd (30) trimester (8% unknown) Postnatal depression: Mother report, CES-D, 12 months Child outcome: Mother report, Brief Infant–Toddler Social and Emotional Assessment, 2 years	Gestational age, birth weight, Apgar score, birth/delivery complications. Did not assess prenatal/postnatal anxiety	Latent growth curve modelling	No	No
Soe et al. [63]	Singapore GUSTO	258 mothers	2 years	Prenatal depression: Mother report, EPDS, 26 weeks. Postnatal depression: Mother report, EPDS, 3 months Child outcome: Mother report, CBCL, 24 months	Ethnicity, prenatal smoking exposure, household income. Did not assess prenatal/postnatal anxiety	Linear regression	No	Yes
van der Waerden et al. [68]	France EDEN	1183 mothers 30.1 years	5 years	Prenatal: Maternal reports, CES-depression, pregnancy Postnatal depression: Maternal reports, EPDS, 4 months, 8 months, 12 months Later depression: Maternal reports, CES-D, 3 years, 5 years Child outcome: Maternal report, SDQ, 5 years	Maternal age, education, prenatal anxiety, substance abuse, use of antidepressants. Low family income, number of siblings, child care arrangements, domestic violence, child sex, premature birth	Growth trajectory analyses, linear regression	No	No
Woolhouse et al. [9]	Australia	1507 30.9 years at birth 60.7% married’ 30.9 years	4 years	Prenatal depression: Mother report, EPDS, 15 weeks Postnatal depression: Mother report, EPDS, 3 months, 6 months, 12 months Later depression: Mother report, EPDS, 4 years Child outcome: Mother report, SDQ, 4 years	Maternal age, country of birth, education level. Relationship and employment status, family income, number of children and relationship transitions since pregnancy. Did not assess prenatal/postnatal anxiety	Mediation analyses	No	Yes

*MD*  Maternal Depression, *Q* quality rating, *EPDS*, Edinburgh Postnatal Depression Scale, *DAWBA*  Development and Wellbeing Assessment, *CBCL* Child Behaviour Checklist, *TRF* Teacher Report Form, *CES* Centre for Epidemiological Studies-Depression Scale, *CIS* Clinical Interview Schedule, *SADS* Schedule for Affective Disorders and Schizophrenia, *SCL* Symptom Checklist, *DSSI* Delusions Symptoms States Inventory, *GAS* Global Assessment Scale, *SDQ* Strengths and Difficulties Questionnaire, *CAPA* Child and Adolescent Psychiatric Assessment, *YSR* Youth Self Report, *BDI* Beck Depression Inventory, *BITSEA* Brief Infant–Toddler Social and Emotional Assessment, *ALSPAC* Avon Longitudinal Study of Parents and Children, *SLCDS* South London Child Development Study, *CD* conduct disorder, *ODD* Oppositional Defiant Disorder, *ADHD* Attention Deficit Hyperactivity Disorder, *DBD* Disruptive Behavioural Disorders (DSM-IV Conduct disorder/Oppositional Defiant Disorder)

^a^Information on maternal age, ethnicity, relationship status and socioeconomic status was not reported in all studies

### Design

By nature of the inclusion criteria all included studies were prospective longitudinal designs, with one study also embedded within a larger randomised controlled trial [60]. The families were followed longitudinally with the shortest follow up when the offspring were 24 months and the longest when the offspring were 16–17 years old. More specifically, the majority of studies (*n* = 10) followed offspring to the early childhood period [9, 43, 49, 60–63, 66, 68]; just 3 studies followed until the middle childhood period [64, 65, 67]; those going on beyond the middle childhood period tended to come from the same study cohorts; SLCDS [47, 48, 51], Finland studies; [69, 70] and ALSPAC studies [71, 72].

### Participants

The studies had a wide range of sample sizes, ranging from 120 to 14,541 mothers recruited (median = 792 mothers). All studies included community samples, with mothers recruited during pregnancy from hospitals, prenatal clinics or via medical records. Mostly the mothers were white, partnered, and middle class and the samples generally represented the population from which they were drawn (where the features could be extracted).

There were some notable exceptions, with one study specifically recruiting young African American women [43].

Eleven studies reported the mother’s mean age at birth of child; this ranged from 18.3 years [43] to 32.8 years [65].

### Measures

#### Exposure: maternal depression

The majority of studies used self-report questionnaires to assess for depression in mothers (*n* = 17 studies). The most frequently used (*n* = 11 studies) measure of maternal depression was the Edinburgh Postnatal Depression Scale [73], a 10 item self-report measure that has been validated for use in the prenatal and postnatal period [73, 74]. The other predominantly used self-report measure (*n* = 4 studies) was the Centre for Epidemiological Studies depression scale, a validated (e.g. [75, 76]), 20 item, self-report scale. Less well validated measures were also infrequently used; the Delusions-Symptoms State Inventory [77] used by Brennan et al. [49] and the short form Symptom Checklist [78] used by Gjerde et al. [61]. In contrast to the use of self-report measures, three articles from one cohort (SLCDS) used standardised diagnostic interviews to assess depression and also used GPs/psychiatrists to interview and/or confirm the final diagnosis [47, 48, 51].

In terms of the timing of the assessment of depression, the prenatal period assessments were conducted throughout the duration of pregnancy (earliest 14 weeks to the latest 36 weeks). Twelve studies examined depression once during pregnancy; seven studies examined depression twice during pregnancy. The Finnish PREDO cohort study [66] measured depression biweekly through pregnancy up to a total of 14 times. Seven articles measured depression post birth on only one occasion; while the remainder ranged from 2 to 5 follow up assessments. Four studies measured in the postnatal period only [44, 60, 62, 63], 5 studies examined in the later period only [43, 64–66, 68], while 11 studies examined in both the postnatal and later period [9, 47–49, 51, 61, 67, 69–72].

#### Outcomes: child antisocial behaviour

All studies used maternal report to examine child outcomes, these were supplemented with additional informants in a limited number of studies. For instance, teacher report was used in a small number of studies [51, 64, 71] and older children also reported on their own behaviour in certain studies [47, 48, 50, 51]. Self-report questionnaires were used in the majority of cases (*n* = 16) and diagnostic interviews in much fewer studies (*n* = 4). Of the questionnaire measures, the Child Behaviour Checklist (CBCL) was the most frequently used (*n* = 7). The CBCL contains externalising scales (which contains social problems, rule breaking behaviour and aggressive behaviour scales) and internalising scales which were also examined in the majority of cases (*n* = 6). Other measures included: The Brief Infant–Toddler Social Emotional Assessment [79]; Antisocial Symptom Questionnaire [80]; the Rutter Revised Preschool Scales [81]; the Strengths and Difficulties Questionnaire [82]; the Youth Self-Report [83]. These measures tended to examine scales of externalising behaviour and also create diagnostic categories of Disruptive Behaviour Disorders (i.e. Conduct disorder; Oppositional Defiant Disorder). Four studies combined what are typically considered internalising and externalising behaviours into one total problem score [9, 44, 49, 62], rather than looking at these constructs independently.

Research derived from the SLCDS [48, 49, 70] used a notably different approach for the assessment of antisocial behaviour, using a comprehensive diagnostic interview; The Child and Adolescent Psychiatric Assessment (CAPA; [84]). The CAPA was administered by trained assistants and generates clinical diagnoses of Disruptive Behaviour Disorders. The authors in one study [51] also combined items to construct a composite measure in an attempt to assess different aspects of the ASB construct, including the assessment of serious violent behaviour as opposed to normal range aggression or global externalising problems. Furthermore, Hay et al. [48] used police reports of arrests and violent arrests to create the dependent variable.

### Summary of quality of studies

Table 1 shows the quality ratings given for each of the articles. The lowest score given was 12 and the highest was 20 out of 24. All were considered acceptable with 11 studies rated as high quality (a quality rating of 18 and above). All were longitudinal studies (given the nature of the inclusion criteria) and as such were generally well resourced and incorporating a powerful design that allowed for complex analyses including mediation analyses.

Furthermore, they included multiple assessment points and good follow-up periods. Common limitations were those associated with longitudinal designs including the significant attrition that was not always random and the non- representativeness of the samples which sometimes made generalisation difficult (e.g. [60, 61]). Furthermore, the self-report nature of the majority of maternal depression measures reduced quality as this impacted on objectivity scores. Most measures were rated as valid and reliable although there were some exceptions which reduced the quality of those studies (e.g. [49]). In most cases mothers reported on their own behaviour and their children’s behaviour and this limited the objectivity of the assessments and increased the risk of shared method variance. Only 8 of 20 studies controlled for perinatal anxiety symptoms.

### Main findings and synthesis

All 20 studies reported significant associations between maternal depression assessed on at least one-time point and child antisocial behaviour outcomes. Findings are now synthesised by the time period of exposure to maternal depression and offspring stage of development.

### Association between prenatal depression and offspring antisocial behaviour

#### Early childhood

Eleven of 20 studies examined the impact of prenatal depression on offspring antisocial behaviour during early childhood [Table 3]. Of these 11 studies, 7 (64%) found prenatal depression to predict elevated rates of offspring antisocial behaviour after the effects of later maternal depression and specified covariates were taken into account (using different statistical and conceptual approaches). One of these studies examined the impact of prenatal depression on offspring antisocial behaviour from early childhood and into adolescence [72] 1. This study found persistently higher offspring behavioural problems following exposure to prenatal depression with no diminishment of effect into adolescence [72]. However, the specific effects of post-pregnancy episodes of maternal depression could not be delineated in this study due to the hypotheses tested and the analytical strategy employed [72]. In general, there did not appear to be clear distinctions between those studies that found an effect of prenatal depression versus those that did not, although there was a trend for those studies that failed to find an association to use more global measures of offspring behavioural problems that included symptoms of emotional problems.

**Table 3:** Study findings and synthesis

Study	Primary results				Mediation/moderation analyses	Main limitations
	Prenatal depression	Postnatal depression (birth to 12 months)	Beyond perinatal depression (after 12 months)	Recurrence/chronicity/cumulative exposure		
Barker et al. [64]	Prenatal MD independently predicted offspring behavioural problems after adjustment for prenatal anxiety, later MD and covariates. No interaction between offspring gender and MD	N/A	Later MD (1.5 years) predicted offspring behavioural problems independently (of prenatal) predicted externalising. No interaction between offspring gender and MD	N/A	N/A	Sample: Low rates of ethnic minorities. Substantial attrition over time Measures: Maternal report of depression and child outcome. Depression not measured concurrently Other: Only included 1 assessment of later maternal depression
Brennan et al. 2000 [49]	Prenatal MD did not predict offspring behavioural problems after adjustment for covariates and MD at other time points. No evidence of gender differences	Postnatal MD predicts elevated offspring total problems after adjustment for covariates and MD at other time points. No evidence of gender differences	Concurrent MD predicts elevated offspring total problems after adjustment for covariates and MD at other time points. No evidence of gender differences	Chronicity and severity of MD predicts elevated offspring behavioural problems after adjustment for covariates and MD at other time points. No evidence of gender differences	N/A	Sample: Lower SES than the population. Non-random attrition (those lost differed in a number of ways from those retained) Measures: Maternal report of depression and child outcome. Other: Child outcome did not distinguish between behavioural and emotional problems. Only included 1 assessment of later maternal depression (concurrent)
Edwards and Hans [43]	The initial association between prenatal MD and offspring behavioural problems was mediated by maternal sensitivity and concurrent maternal MD. Effects only significant for boys	N/A	The initial association between prenatal MD and offspring behavioural problems was mediated by maternal sensitivity and concurrent maternal MD. Effects only significant for boys	N/A	Maternal sensitivity mediates the relationship between prenatal MD and offspring behavioural problems	Sample: Small homogenous sample not representative of general population Measures: Maternal report of depression and child outcome. Other: Outcome did not distinguish between behavioural/emotional problems. Only included 1 assessment of later maternal depression (concurrent)
Eichler et al. [65]	Prenatal MD predicts offspring antisocial behaviour after controlling for current MD and covariates. Effects significant for boys but not girls	N/A	Concurrent MD (child 6-9 years) did not predict offspring antisocial behaviour after adjustment for covariates and prenatal MD	N/A	N/A	Sample: Significantly higher education, makes generalisation difficult. Relatively small sample size Measures: Mother self-report on depression and child outcome Other: Only included 1 assessment of later maternal depression (concurrent)
Gjerde et al. [61]	Prenatal MD does not predict offspring behavioural problems after adjustment for unmeasured confounding. Gender differences not tested	Postnatal MD does not predict offspring behavioural problems after adjustment for unmeasured confounding	Concurrent MD predicts elevated offspring behavioural problems after adjustment for unmeasured confounding	N/A	Unmeasured familial confounding explains initial association between prenatal MD and offspring behavioural problems	Sample: Substantial attrition Measures: Mother self-report on depression and child outcome Other: Only included 1 assessment of later maternal depression (concurrent)
Hanington, et al. [44]	Prenatal MD (2nd trimester) and marital conflict during pregnancy predicted elevated offspring conduct problems when postnatal MD and paternal pre-and-postnatal depression were accounted for. Gender differences not tested	Postnatal MD (8 months) predicted elevated offspring conduct problems when prenatal MD, paternal pre-and-postnatal depression, and marital conflict were accounted for	N/A	N/A	Marital conflict partially mediated the relationship between postnatal MD and offspring conduct problems	Sample: Low rates of ethnic minorities. Substantial attrition over time Measures: Mother self-report on depression and child outcome Other: Does not include measures of later maternal depression
Hay et al. (2003) [51]	Prenatal MD did not predict offspring violence after subsequent episodes of MD and covariates were adjusted for. Boys more likely to show violence than girls	Postnatal MD predicted offspring violence after adjustment for prenatal MD, later MD and covariates. Boys more likely to show violence than girls	Current psychological distress (GAS Score) associated with child violence but did not explain the impact of postnatal MD	Risk for violence was greatest in the group of children whose mothers were depressed in the postpartum period and at least once thereafter	N/A	Sample: Small sample size drawn from a large metropolitan area and more ethnically diverse than the general population Measures: Retrospective reports of maternal depression between developmental time periods
Hay et al. (2010) [48]	Prenatal MD significantly predicted offspring violence after controlling for covariates and exposure to subsequent episodes of MD. Boys and girls were not significantly different in ASB or violence specifically	Postnatal MD did not predict offspring violence after covariates and maternal MD at other time points was taken into account. Boys and girls were not significantly different in ASB or violence specifically	Exposure to MD during either early, middle or late childhood did not predict offspring ASB once the impact of prenatal MD was accounted for	Recurrent exposure to maternal MD predicted offspring violence but did not explain the effect of prenatal depression	N/A	Sample: Small sample size drawn from a large metropolitan area and more ethnically diverse than the general population Measures: Retrospective reports of maternal depression between developmental time periods
Hay et al. (2008) [47]	Prenatal MD did not predict elevated risk of offspring disruptive behavioural disorders. No significant differences between boys and girls in risk of disruptive behavioural disorders	Postnatal MD did not predict elevated risk of offspring disruptive behavioural disorders. No significant differences between boys and girls in risk of disruptive behavioural disorders	Exposure to maternal depression after 3 months postpartum predicted elevated rates of offspring DBD. Exposure to later MD not analysed at the level of specific time points	Cumulative exposure to maternal depression after the perinatal period (during early to late childhood and/or adolescence) predicted elevated rates of offspring DBD	N/A	Sample: Small sample size drawn from a large metropolitan area and more ethnically diverse than the general population Measures: Retrospective reports of maternal depression between developmental time periods
Korhonen et al. (2012) [70]	Prenatal MD (3rd trimester) predicted offspring reported behavioural problems following adjustment for concurrent MD. Findings only significant for boys. Prenatal MD did not predict maternal reports of offspring behavioural problems	Postnatal MD predicted offspring reported behavioural problems following adjustment for concurrent MD. Findings only significant for male offspring. Postnatal MD did not predict maternal reports of offspring behavioural problems	Concurrent MD predicted elevated offspring behavioural problems according to mothers and adolescents own self-reports. Findings significant for males only	Recurrent (number of times scored over the EPDS cut point) MD predicted elevated offspring behavioural problems. Gender differences not tested due to small cell sizes	N/A	Sample: Number of symptomatic mothers and children was low; Attrition was high. Relatively moderate sample size Measure: Limited control for sociodemographic and other risk factors Other: Only included 1 assessment of later maternal depression (concurrent)
Korhonen et al. (2014) [69]	Prenatal MD predicted elevated offspring behavioural problems prior to controlling for other episodes. Results only significant for mothers’ but not offspring self-reports. Gender differences not tested	Initial exposure to MD at 2 weeks or 6 months postpartum was not associated with elevated offspring behavioural problems for either informant. Gender differences not tested	For both informants, initial exposure to MD at 4–5 and 8–9 years was not associated with offspring behavioural problems. Prior to controlling for other episodes, initial exposure to MD at aged 16–17 (concurrent) years was associated with elevated offspring behavioural problems for self but not maternal reports	Chronic maternal depressive symptoms rather than exposure at specific time periods predict adolescent behavioural problems	N/A	Sample: Number of symptomatic mothers and children was low, attrition is high. Relatively moderate sample size Measure: Self-report measures of depression. Initial exposure to MD was used as a measure (the first time at each time point that a mother exceeded cut off for EPDS, rather than the independent effect)
Lahti et al. [66]	Prenatal MD had a direct effect on offspring externalising behavioural problems. Gender differences not tested	N/A	MD symptoms at the time of the child assessment (1.9 to 5.9 years) partially mediated the effect of prenatal MD on offspring behavioural problems	N/A	N/A	Sample: Homogenous sample, significant attrition Measures: Mother self-report on depression and child outcome Other: Only included 1 assessment of later maternal depression (concurrent)
Leis et al. [71]	Prenatal MD predicted an increase in total offspring conduct problems. Effect remained after controlling for later MD and anxiety for mothers but not teachers’ reports of child behavioural	Postnatal MD predicted increased offspring conduct problems for both mothers and teachers reports at the univariate level. The impact of prenatal depression and other covariates were not examined	N/A	N/A	N/A	Sample: High attrition Measures: Mother self-report on depression and child behaviour Other: The impact of post-pregnancy MD and the specific time periods that MD was measured were not reported/analysed in sufficient detail to extract the required information
Luoma et al. [67]	Prenatal MD predicted elevated offspring behavioural problems for combined maternal and teacher reports. Results remained significant after controlling for subsequent exposure to MD and covariates. Effects stronger for boys than girls	Postnatal MD did not predict offspring behavioural problems	Concurrent MD did not predict offspring behavioural problems. Effects stronger for boys than girls	N/A	N/A	Sample: Moderate-sized sample, small group sizes for MD Measures: Mother self-report on depression and child outcomes Other: Results are not clearly reported. Only included 1 assessment of later maternal depression (concurrent)
O’Connor et al. [62]	Prenatal MD does not predict offspring behavioural problems after the influence of prenatal anxiety is accounted for. No gender differences	Postnatal depression at 8-weeks and 8-months postpartum predicts offspring behavioural problems after controlling for prenatal depression and anxiety and covariates. No gender differences	N/A	N/A	N/A	Sample: Substantial Attrition Measures: All data based on maternal reports Other: Does not distinguish between child emotional/behavioural problems. Did not include an assessment of later maternal depression
O’Donnell et al. [72]	Prenatal MD predicted elevated offspring behavioural/emotional problems. The effects were observed after controlling for multiple confounders including prenatal anxiety and postnatal depression. No interaction between MD exposure and offspring gender	Unobtainable	Unobtainable	N/A	N/A	Measures: All data based on mother reports Other: Results were not reported in sufficient detail to extract the required information e.g. cannot distinguish post-pregnancy depression data. Did not distinguish child behavioural and emotional problems
Raskin et al. [60]	Prenatal MD predicted elevated offspring behavioural problems following adjustment for later MD and covariates. Gender differences not tested	N/A	MD between 1 and 2 years predicted elevated offspring behavioural problems following adjustment for prenatal MD and covariates. Gender differences not tested	Chronic MD from pregnancy to 2 years predicted elevated offspring behavioural problems. Gender differences not tested	N/A	Sample: High risk sample accessing a home visiting programme which limits generalizability Measures: Mother self-report on depression and child outcome. Potential shared method variance Other: Did not distinguish between child behavioural/emotional problems
Soe et al. [63]	Prenatal MD predicted elevated offspring behavioural problems following adjustment for covariates. Findings only significant for females	Postnatal MD predicted elevated offspring behavioural problems following adjustment for covariates. Findings only significant for females	N/A	N/A	N/A	Sample: High attrition Measures: Mother self-report on depression and child outcome. Potential shared method variance Other: Did not include a measure of later MD
van der Waerden et al. [68]	Prenatal MD did not predict elevated offspring behavioural problems following adjustment for covariates and later MD. Gender differences not reported	Postnatal MD predicted elevated offspring behavioural problems following adjustment for covariates and concurrent MD at 5 years. Gender differences not reported	Exposure to MD during the preschool years predicted elevated offspring behavioural problems following adjustment for covariates and concurrent MD at 5 years. Gender differences not eported	Exposure to chronic (persistent) MD from infancy and throughout early childhood predicted elevated rates of offspring behavioural problems. Gender differences not reported	N/A	Sample: Attrition not random (women depressed during pregnancy more likely to drop out) Measures: All data based on mother reports Other: Concurrent MD included as a covariate did not explore/report independent effect
Woolhouse et al. [9]	Prenatal MD predicts elevated offspring behavioural/emotional problems at the univariable level. Only combined exposure to prenatal and postnatal MD significant after adjustment for covariates. Boys had greater behavioural/emotional problems than girls	Postnatal MD predicts elevated offspring behavioural/emotional problems at the univariable level. Only combined exposure to postnatal and prenatal MD significant after adjustment for covariates. Boys had greater behavioural/emotional problems than girls	MD (concurrent) at 4 years postpartum had an independent effect on child behavioural/emotional problems after adjusting for prenatal and postnatal MD and covariates. Boys had greater behavioural/emotional problems than girls	N/A	N/A	Sample: Young women, single women and non-English speaking women are underrepresented. Selective attrition Measures: Maternal self-report of depression and child outcome Other: Did not distinguish between child behavioural/emotional problems. Only included 1 measure of later MD (concurrent)

Four of 11 studies that assessed offspring ASB during early childhood did not find significant associations between prenatal depression and child ASB following adjustment for covariates and subsequent episodes of maternal depression [49, 61, 62, 69]. Brennan et al. [49] found that behavioural problems were higher for children whose mothers were depressed only at 6 months postpartum, or at 5 years (compared to those during pregnancy or at birth). The measure used to assess maternal depression in this study was brief and not well established and the quality score was relatively low compared to the other studies (14 out of 24). Similarly, O’Connor et al. [62] also failed to find an association between prenatal depression and offspring ASB (while prenatal anxiety was associated). It is noteworthy that both of these studies used combined measures of behaviour problems that included aspects of emotional problems (rather than a distinct or ‘pure’ measure of antisocial behaviour).

#### Middle to late childhood

Five studies followed-up children during middle [64–66] or late childhood [51, 72]. Four of these studies (80%) found prenatal depression to predict elevated child ASB. These findings remained significant following adjustment for potential confounds and subsequent episodes of maternal depression. The one study that did not find prenatal depression to increase the risk of offspring ASB tested pre-and-postnatal depression within the same statistical model—finding postnatal but not prenatal depression to be the significant predictor of offspring ASB [51].

#### Adolescence

Four of 20 studies followed-up children in adolescence [47, 48, 69, 70] with one study using an outcome variable of disruptive behaviour disorders that combined assessments at 11 and 16 years [47]. This latter study did not find a significant association between exposure to prenatal depression and offspring ASB. Rather, cumulative exposure to maternal depression from infancy to adolescence increased the risk of offspring ASB in this study [47]. Thus, 3 of 4 studies (75%) found prenatal depression to be a risk factor for offspring ASB in late adolescence (16–17 years of age)—with the results remaining significant after statistical control for covariates and later episodes of maternal depression. It is noteworthy that each of these three studies included offspring as well as mothers’ as informants on the ASB outcome.

### Association between maternal postnatal depression and offspring antisocial behaviour

#### Early childhood

Seven of 20 studies examined the impact of postnatal depression (0–12 months postpartum) on offspring ASB during early childhood. Six of these 7 studies (86%) found exposure to postnatal depression to increase the risk of offspring ASB during early childhood [9, 44, 49, 60, 62, 63, 73]. However, 3 of these studies did not control for the impact of exposure to maternal depression after the perinatal period on offspring risk of ASB [44, 62, 63] and in one study, only exposure to both prenatal and postnatal maternal depression  predicted offspring ASB [9]. In this latter study exposure to either prenatal or postnatal depression alone did not confer risk for offspring ASB. Given the lack of control for exposure to maternal depression after the perinatal period findings of positive associations between postnatal depression and offspring risk of ASB should be viewed with caution.

#### Middle-to-late childhood

Three of 20 studies examined the impact of postnatal depression on offspring ASB during middle-to-late childhood [51, 67, 71]. Each of these studies used multiple informants of offspring ASB (eithers teachers or the child themselves). The findings are mixed. One study did not find postnatal depression to be a risk factor for offspring behavioural problems [67]. One study found exposure to postnatal depression was a risk factor for offspring violence at 11 years after the influence of covariates, prenatal depression and later maternal depression was taken into account [51]. Similarly, the study by Leis and colleagues also found postnatal depression to be a significant risk factor for offspring behavioural problems. However, in this study, the significance of this association was not tested with covariates and later maternal depression in the same statistical model [71]. Thus, evidence is limited for a unique association between postnatal depression and offspring ASB in middle-to-late childhood.

#### Adolescence

Four of 20 studies examined the impact of postnatal depression on offspring ASB in adolescence [47, 48, 69, 70], with one of these studies using an outcome variable of disruptive behaviour disorders that combined assessments at 11 and 16 years [47]. Two of these studies used symptom checklists of behavioural problems using both mothers and the adolescents themselves as informants [69, 70]. The other study used diagnoses of conduct disorder and the adolescents arrest history to construct two outcome variables, ASB with and without violence [48]. Only one study found postnatal depression to increase the risk of offspring ASB [70]. In this study, postnatal depression predicted adolescent's, but not mother’s reports of offspring ASB with this effect only significant for males. The other 3 studies failed to find an association between postnatal depression and offspring ASB following control for covariates and maternal depression at other time points.

### Association between later maternal depression and offspring antisocial behaviour

#### Early childhood

Eight of 20 studies examined the impact of exposure to maternal depression after the perinatal period on offspring ASB in early childhood. Six of these studies measured maternal depression concurrently to the assessment of offspring ASB [9, 43, 60, 61, 64, 66]. Two of these studies used a statistical approach (trajectory analyses) that did not allow for the separation of exposure to later maternal depression into different time periods [68, 72]. All of these studies found exposure to maternal depression after the perinatal period increased the risk of offspring ASB after the influence of covariates and prior maternal depression were taken in account. Four studies found prenatal and later exposure to maternal depression to independently predict an elevated risk of offspring ASB [9, 60, 64, 72]. Two studies found exposure to later maternal depression to partially mediate the relationship between prenatal depression and offspring ASB [43, 66]. Whereas two studies found that it was exposure to later maternal depression and not prenatal depression that conferred risk [61, 68].

#### Middle-to-late childhood

Four studies examined the impact of later maternal depression on offspring ASB in middle-to-late childhood [47, 60, 61, 63]. The majority of these studies included a measure of concurrent maternal depression [51, 64, 67]. Two of these studies found that it was prenatal depression and not later depression that increased the risk of offspring ASB [65, 67]. Whereas two studies found that exposure to maternal depression during pregnancy and after the perinatal period independently increased the risk of offspring ASB [51, 64].

#### Adolescence

Four of 20 studies included an assessment of exposure to later maternal depression and offspring ASB in adolescence [47, 48, 69, 70]. Three studies found exposure to maternal depression after the perinatal period to increase the risk of offspring ASB [47, 69, 70], with two of these studies using a measure of concurrent maternal depression [69, 70]. One study found that it was exposure to prenatal and not later maternal depression that placed offspring at risk of ASB in adolescence [48]. In this study, Hay and colleagues examined the impact of exposure to later maternal depression on offspring ASB during different developmental time periods (early, middle, and late childhood). It is noteworthy that the four studies that followed children up into adolescence were drawn from two longitudinal cohorts (the South London Child Development Study in the UK and the Tampere cohort from Finland.

### Association between cumulative exposure to maternal depression and offspring ASB

#### Early-to-late childhood

Four studies examined the impact of cumulative exposure to maternal depression on offspring ASB. Three of these studies assessed offspring ASB during early childhood [49, 60, 68] and one during late childhood [51]. All of these studies found cumulative exposure to maternal depression to increase the risk of offspring ASB following the statistical control for covariates. In two of these studies cumulative exposure to maternal depression during early childhood predicted offspring ASB, whereas prenatal depression did not [49, 68]. In one study cumulative exposure to maternal depression from pregnancy to 2 years predicted offspring ASB [60]. In another study, cumulative exposure to maternal depression from the postnatal period and throughout childhood conferred the greatest risk [51]. Prenatal depression was not a predictor of offspring ASB in this study [51]. Overall, cumulative exposure to maternal depression was shown to be more important than exposure at one specific time period during early childhood.

#### Adolescence

Four studies examined the impact of cumulative exposure to maternal depression on offspring ASB during adolescence [47, 48, 69, 70]. Three of these studies reported cumulative exposure to maternal depression through childhood and into adolescence to confer the greatest risk for offspring ASB. One study found that it was exposure to prenatal and not later maternal depression that placed adolescent offspring at greatest risk of ASB [48]. It should be noted that variables related to cumulative depression were termed, defined and operationalised in diverse ways making comparison difficult. Overall, the evidence pointed towards mothers experiencing depression on multiple occasions and that this cumulative exposure conferred the greatest risk for offspring ASB, particularly when this exposure began during the perinatal period.

### Mechanisms underlying the association between maternal depression and offspring ASB

The majority of studies focussed on exploring how exposure to maternal depression at different time points conferred risk for offspring ASB. Only three of 20 studies explored biological or environmental mechanisms that could explain the identified associations [43, 44, 61]. Edwards and Hans [43] found that the association between prenatal depression and toddler behaviour problems was mediated by maternal sensitivity as well as maternal depressive symptoms at 24 months. In another study marital conflict partially mediated the relationship between postnatal depression and offspring behavioural problems [44]. Finally, the study by Gjerde and colleagues found that after adjusting for unmeasured familial confounding within a genetically informative design, the association between prenatal depression and offspring behavioural problems was no longer significant [61]. Whilst conclusions are limited by the small number of studies, it is clear that when hypothesised mechanisms underpinning the relationship between maternal depression and offspring ASB are examined, the direct effect of prenatal depression is significantly diminished.

### Gender differences

Twelve of 20 studies tested for gender differences. Five of these 12 studies (42%) found boys to be more vulnerable to the effects of maternal depression [43, 51, 65, 67, 70] with one study reporting females to be at greater risk [63]. For instance, Edwards and Hans [43] found that associations between prenatal depression, maternal sensitivity, and behaviour problems were especially strong among boys. Korhonen et al. [70] found sex differences depended on timing—concurrent maternal depression was associated with adolescent behavioural problems for both genders, whereas maternal prenatal and postnatal depressive symptoms were associated with behavioural problems for boys only. In contrast, Hay et al. [48] did not find gender differences in their analyses of prenatal and cumulative exposure to maternal depression and offspring ASB.

## Discussion

This review systematically evaluated longitudinal studies that examined exposure to maternal depression both during pregnancy and at least once post birth and offspring risk of antisocial behaviour. Twenty studies met the inclusion criteria and were systematically reviewed. Only three studies examined hypothesised biological or environmental mechanisms that could underpin associations between maternal depression and offspring ASB. There was evidence that prenatal, postnatal and later episodes of maternal depression were all predictive of antisocial outcomes. One particular time period of maternal depression exposure did not emerge as relatively more important in the prediction of offspring ASB than another. However, measures of exposure to maternal depression after the perinatal period were limited and typically included a one-off assessment of mothers’ depressive symptoms—often measured concurrently to the assessment of offspring ASB. Therefore, exposure to maternal depression after the perinatal period but before the concurrent assessment of offspring ASB could explain the documented associations. Indeed, when cumulative exposure to maternal depression and specific timing effects were measured within the same study (*N* = 8 studies), it was cumulative exposure that conferred the greatest risk for offspring ASB—particularly when this exposure began during the perinatal period. Only 1 of 8 studies found specific timing effects of prenatal depression when the impact of cumulative exposure was accounted for [48].

The results of individual studies were highly varied, using diverse analytical approaches and not all studies explored the independent effects of exposure to maternal depression during different stages of child development. The range of covariates tested across the included studies were not consistent, and only 8 of 20 studies controlled for prenatal anxiety symptoms. Thus, associations between maternal depression and offspring ASB could be artificially inflated in 12 studies as the effects of perinatal anxiety symptoms were not accounted for. Many of the studies came from the same larger cohorts with just 10 longitudinal cohorts identified in total. Although it is likely that additional studies exist and were not included in this synthesis, this remains a relatively small number of longitudinal studies exploring the important associations between maternal depression and offspring ASB. The ALSPAC and SLCDS cohorts dominated the review findings and both are from England and therefore may not be generalizable to other countries. With the ALSPAC cohort in particular there was significant attrition and the subsamples used in a given study may not be representative. Furthermore, within the same cohort study, exposure to prenatal, postnatal, and later maternal depression was found to differ in conferring risk for offspring ASB depending on the age of the child, the outcome measure, the informant, the analytical strategy, and the control for exposure to maternal depression at other time points. Given the methodological and conceptual limitations with the included studies it is argued that further research that includes tests of underpinning biological and environmental mechanisms is required to disentangle the complex associations between maternal depression and offspring antisocial behaviour.

A general limitation of this literature is that mothers reported on both their own depressive symptoms and their child’s antisocial behaviour with few studies including additional informants (e.g. teachers/children) or using more objective assessment measures (e.g. diagnostic interviews). The mother reporting on both the independent and dependent variable could artificially inflate effect sizes. There is some evidence that depressed and/or anxious mothers have less accurate perceptions of their child’s difficulties and may over report problems (e.g. [85–87]). Nevertheless, other evidence suggests that the associations remain independent of the effects of maternal depression on maternal reporting errors [85, 87]. Given the complex picture, future research should include multiple informants on child behaviour and more objective diagnostic interviews of the mother’s mental health would strengthen the evidence base and potentially help clarify the picture further.

It is important to note that despite the range of different measures of antisocial behaviour used among the included studies there were few that went beyond looking at broadband constructs such as behavioural (*externalising*) problems, or at the more severe end of the spectrum, disruptive behaviour disorders. Most studies did not look at the subtleties of ASB (e.g. violence, intentional aggression) and its different domains. ASB is a heterogeneous construct and attempts have been made to examine subgroups that may have different underlying causal pathways [88]. The use of varied conceptualisations of ASB would be useful in future studies to more fully explore associations with exposure to maternal depression. Future studies would also benefit from being more specific in their measurement of ASB and following children up beyond early childhood. A small number of studies included in this review used an ASB outcome measure that incorporated symptoms of emotional problems and this approach could conceal important effects unique to ASB. Developmentally appropriate measures of specific domains of ASB (e.g. reactive and proactive aggression in early childhood or conduct problems with/without violence) could help clarify associations, aid in the search for underlying mechanisms, and lead to more targeted intervention programmes.

Depression is also heterogeneous and this review has not examined factors such as the severity and duration of symptoms which could influence outcomes in different ways [49]. The vast majority of included studies used symptom rating scales of depressive symptoms rather than diagnostic assessments of maternal depression. A general challenge in the literature is disentangling the effects of severity and duration of exposure to maternal depression as well as specific timing effects. As mentioned previously, only 8 studies examined the effects of cumulative exposure, and assessments of maternal depression after the perinatal period were typically confined to one-off measures of depressive symptoms assessed concurrently to offspring ASB. Studies that invested in depth of measurement (e.g. the use of clinical interviews and diagnoses in the SLCDS) over sample size still relied on mothers’ recalling episodes of depression over a two-to-five-year period. As such, we cannot be certain on the duration of the child’s exposure to symptoms of maternal depression between assessments. Future research should improve upon these methodological limitations and include multiple assessments of maternal depression that test severity and duration of exposure as well as specific timing effects.

The evidence from this review suggests that there are independent effects of exposure to maternal depression during pregnancy, the postnatal period, and throughout childhood on offspring ASB. However, given the aforementioned methodological limitations these conclusions are only tentative. Cumulative exposure to maternal depression, with the initial exposure often beginning in pregnancy is indicated as being particularly detrimental. It is not within the scope of this review to disentangle the highly complex potential causal pathways of the association between maternal depression exposure and offspring ASB. The effects of the prenatal period may imply biological pathways affecting the foetus directly, genetic transmission, epigenetic mechanisms and/or maternal programming effects [19, 20, 29, 41, 42]. Whereas exposure to maternal depression during the postnatal period may influence the quality of the care-giving environment and the parent–child relationship [34–36]. Exposure to maternal depression beyond the perinatal period may confer additional risk for offspring ASB through parental modelling of the cognitive, affective and behavioural aspects of depression and impaired or inadequate parenting strategies [36, 46]. The findings of this review call for future studies to examine the interacting etiological mechanisms that are likely operating from the prenatal period and throughout childhood and adolescence in the context of maternal depression exposure. Longitudinal designs that allow for the estimation of genetic risk are also required as it is likely that associations between maternal depression and offspring ASB are overestimated in studies that do not account for unmeasured familial confounding [61].

Longitudinal designs are powerful and allow pathways to be examined, but correlation does not imply causation and the included studies cannot assess the causal relations between maternal depression and offspring ASB. The observed associations likely reflect a multifactorial process that includes biological and environmental mechanisms. Indeed, the three studies that did examine potential mechanisms drew attention to genetic and environmental (maternal sensitivity/inter-parental conflict) processes [43, 44, 61]. Finding that once their influence was taken into account, the magnitude of the association between perinatal depression and offspring ASB was considerably reduced. Future research needs to extend these findings and focus on the different underlying mechanisms hypothesised to be operating during the different time periods of a child’s development.

### Strengths and limitations of the systematic review

The findings of the review are the result of a rigorous, systematic attempt to synthesise a large body of research. Systematic criteria (i.e. PRISMA) were used to identify studies and a quality assessment tool was used to critically appraise the studies. Nonetheless, the findings must be considered in view of a number of limitations; only English articles were included therefore running the risk that important articles published in other languages may have been missed. In addition, unpublished studies were excluded in the search process which runs the risk of the file drawer problem and the studies being a biassed representation. However, grey literature including abstracts, conference proceedings and governmental reports, were examined if relevant and provided useful supplementary information for overall arguments. There did not appear to be an indication of a bias where unpublished, grey literature was yielding a different pattern of findings.

We did not apply the quality rating system to the broader literature summarised in the introduction. Whilst we did select well cited reviews and empirical studies often conducted by eminent scientists in the field, it could be argued that this literature contains biases that we have overlooked. Within the available resources we did aim to reduce and highlight potential sources of bias. For example, a second independent researcher examined and rated the quality of the included studies, and a second researcher independently reviewed the 20 studies and checked the extraction of the necessary information. However due to limited resources, a second researcher did not repeat the entire search strategy from start to finish.

### Future direction and recommendations

Several recommendations relating to research, clinical practice, and policy development arise from this systematic review.

### Research recommendations

As alluded to, further research is required that includes multiple informants on the measures of interest rather than the mother as sole informant on the child’s behaviour. More objective, observable measures of ASB as well as maternal depression would be a useful addition to research in this area. Further exploration of mechanisms of effect would also increase understanding and point to potential avenues for intervention. In line with developments in the animal literature, associations between maternal prenatal depression and offspring ASB could in part be explained by *paternal programming* effects including the impact of sperm quality, seminal plasma and sperm mediated epigenetic mechanisms on the maternal reproductive system [89, 90]. For example, seminal plasma has been shown to modify the cell signalling, vascular and nutrient environment of the maternal reproductive tract following conception which impacts on early embryo development [90]. Furthermore, paternal lifestyle factors (e.g. diet, obesity) have been linked with modifications to the early maternal uterine environment that impact on offspring development [89, 90]. Future studies also need to include more comprehensive measures of maternal depression after the perinatal period. ASB is heterogeneous and although in the wider literature it has been explored in diverse ways, this development has not transferred into the longitudinal studies examining the impact of maternal depression on offspring ASB [92]. Future research should attempt to explore how maternal depression (including indices of severity and duration of exposure) at different time points relates to different aspects of antisocial behaviour as this may facilitate the development of more targeted and effective interventions.

### Clinical/policy recommendations

The evidence from this review demonstrates that maternal depression at each stage of motherhood has an impact on child development and this begins early from at least the prenatal period. Prenatal maternal depression is associated with adverse offspring outcomes that persist into adolescence—even when subsequent episodes and covariates are taken into account (e.g. [73]). We also know that intervention for child antisocial behaviour is often ineffective in later years and early intervention is imperative [87]. The findings from the review provide further support for early detection of difficulties and intervention for both mothers and their children. This is consistent with NICE guidance [91] for prenatal and postnatal mental health that argues for early detection and management of mental health during pregnancy and in the first year postnatally. Many women still do not have access to perinatal mental health services with a cost of an estimated £8.1 billion for each one-year cohort of births in the UK [4]. This review provides additional support for the importance of providing services during this time.

The findings also advocate for the importance of continued monitoring and support for mothers and their children beyond the perinatal period. There were independent associations between maternal depression and child ASB beyond the first year of life and some studies find higher rates of depression outside of the perinatal period [9]. Woolhouse and colleagues [9] argue for the rethinking of current policy frameworks as many women may fall through the gaps as they do not reconnect with mental health services beyond the perinatal period. Finally, the evidence also suggests that for some women depression is recurrent and this cumulative exposure is particularly problematic for children. Unfortunately, it is not clear to what extent mothers in these studies had received treatment for depression during the perinatal period, nevertheless the evidence would suggest that some mothers need intervention early specifically on how to manage chronic illness rather than advice on the short term reduction of their current difficulties [47].

## Conclusions

Prenatal, postnatal, and later episodes of depression are all predictive of antisocial outcomes. However, correlation does not equate to causation. When cumulative exposure to maternal depression and specific timing effects were measured within the same study it was cumulative exposure that conferred the greatest risk for offspring ASB—particularly when this exposure began during the perinatal period. Studies that have investigated the association between exposure to maternal depression from pregnancy and throughout childhood are small in number, rarely account for the influence of shared method variance, are often based on the same cohort, and examine a relatively narrow conceptualisation of the antisocial behaviour construct. Further research is required that seeks to improve on these methodological limitations and examine the biological, psychological and environmental mechanisms that mediate and moderate the observed associations.

## Electronic supplementary material

Below is the link to the electronic supplementary material.

Supplementary material 1 (DOCX 32 kb)

## Footnotes

1 The main findings of this study will be summarised here. It is important to note that prenatal depression was found to increase the risk of offspring behavioural problems across developmental time periods during childhood and adolescence.
