# Supplementary material for: Longitudinal studies examining the impact of prenatal and subsequent episodes of maternal depression on offspring antisocial behaviour
Source: Eur Child Adolesc Psychiatry. 2019 Dec 2;30(1):5–40. doi: 10.1007/s00787-019-01447-w (PMC7864821; doi:10.1007/s00787-019-01447-w)
Supplement: Supplementary file 1 — Supplementary material 1 (DOCX 32 kb) [file 787_2019_1447_MOESM_ESM.docx]

Appendix 1. Excluded studies following review (N = 139 studies)

Abulizi, X., Pryor, L., Michel, G., Melchior, M., & van der Waerden, J. (2017). Temperament in infancy and behavioral and emotional problems at age 5.5: The EDEN mother-child cohort. *PLoS ONE*, *12*(2). Retrieved from http://ovidsp.ovid.com/ovidweb.cgi?T=JS&PAGE=reference&D=psyc13&NEWS=N&AN=2017-07593-001

Allen, N. B., Lewinsohn, P. M., & Seeley, J. R. (1998). Prenatal and perinatal influences on risk for psychopathology in childhood and adolescence. *Development and Psychopathology*, *10*(3), 513–529. https://doi.org/http://dx.doi.org/10.1017/S0954579498001722

Ammaniti, M., Sergi, G., Speranza, A. M., Tambelli, R., & Vismara, L. (2002). High-risk motherhood, early interactions, and attachment behavior in infancy. *Maternita a Rischio, Interazioni Precoci E Attaccamento Infantile.*, *72*, 61–67. Retrieved from http://ovidsp.ovid.com/ovidweb.cgi?T=JS&PAGE=reference&D=psyc4&NEWS=N&AN=2002-17592-006

Anderson, A. K. (2014). Prenatal maternal depression and the neural development of social cognition. *Dissertation Abstracts International: Section B: The Sciences and Engineering*, *74*(11–B(E)), No-Specified. Retrieved from http://ovidsp.ovid.com/ovidweb.cgi?T=JS&PAGE=reference&D=psyc11&NEWS=N&AN=2014-99100-551

Anderson, H. (2007). Maternal risk factors and child behavior problems. *Dissertation Abstracts International: Section B: The Sciences and Engineering*, *67*(10–B), 6092. Retrieved from http://ovidsp.ovid.com/ovidweb.cgi?T=JS&PAGE=reference&D=psyc5&NEWS=N&AN=2007-99008-292

Augustine, J. M., & Crosnoe, R. (2010). Mothers’ depression and educational attainment and their children’s academic trajectories. *Journal of Health and Social Behavior*, *51*(3), 274–290. https://doi.org/http://dx.doi.org/10.1177/0022146510377757

Azar, R., Paquette, D., Zoccolillo, M., Baltzer, F., & Tremblay, R. E. (2007). The association of major depression, conduct disorder, and maternal overcontrol with a failure to show a cortisol buffered response in 4-month-old infants of teenage mothers. *Biological Psychiatry*, *62*(6), 573–579. https://doi.org/http://dx.doi.org/10.1016/j.biopsych.2006.11.009

Babineau, V., Green, C. G., Jolicoeur-Martineau, A., Bouvette-Turcot, A.-A., Minde, K., Sassi, R., … Wazana, A. (2015). Prenatal depression and 5-HTTLPR interact to predict dysregulation from 3 to 36 months-A differential susceptibility model. *Journal of Child Psychology and Psychiatry*, *56*(1), 21–29. https://doi.org/http://dx.doi.org/10.1111/jcpp.12246

Bandoli, G., Coles, C. D., Kable, J. A., Wertelecki, W., Granovska, I. V, Pashtepa, A. O., & Chambers, C. D. (2016). Assessing the independent and joint effects of unmedicated prenatal depressive symptoms and alcohol consumption in pregnancy and infant neurodevelopmental outcomes. *Alcoholism: Clinical and Experimental Research*, *40*(6), 1304–1311. https://doi.org/http://dx.doi.org/10.1111/acer.13081

Barker, E. D. (2013). The duration and timing of maternal depression as a moderator of the relationship between dependent interpersonal stress, contextual risk and early child dysregulation. *Psychological Medicine*, *43*(8), 1587–1596. https://doi.org/https://dx.doi.org/10.1017/S0033291712002450

Barker, E. D., Oliver, B. R., Viding, E., Salekin, R. T., & Maughan, B. (2011). The impact of prenatal maternal risk, fearless temperament and early parenting on adolescent callous-unemotional traits: A 14-year longitudinal investigation. *Journal of Child Psychology and Psychiatry and Allied Disciplines*, *52*(8), 878–888. https://doi.org/10.1111/j.1469-7610.2011.02397.x

Barker, E. D., Copeland, W., Maughan, B., Jaffee, S. R., & Uher Rudolf; ORCID: http://orcid.org/0000-0002-2998-0546, R. A. I.-O. http://orcid. org/Uhe. (2012). Relative impact of maternal depression and associated risk factors on offspring psychopathology. *The British Journal of Psychiatry*, *200*(2), 124–129. https://doi.org/http://dx.doi.org/10.1192/bjp.bp.111.092346

Barker, E. D., Kirkham, N., Ng, J., & Jensen, S. K. G. (2013). Prenatal maternal depression symptoms and nutrition, and child cognitive function. *The British Journal of Psychiatry*, *203*(6), 417–421. https://doi.org/http://dx.doi.org/10.1192/bjp.bp.113.129486

Barker, E. D., & Maughan, B. (2009). Differentiating Early-Onset Persistent Versus Childhood-Limited Conduct Problem Youth. *AMERICAN JOURNAL OF PSYCHIATRY*, *166*(8), 900–908. https://doi.org/10.1176/appi.ajp.2009.08121770

Barthel, D., Kriston, L., Fordjour, D., Mohammed, Y., Kra-Yao, E. D., Bony Kotchi, C. E., … Ehrhardt, S. (2017). Trajectories of maternal ante- and postpartum depressive symptoms and their association with child- and mother-related characteristics in a West African birth cohort study. *PLOS ONE*, *12*(11), e0187267. https://doi.org/10.1371/journal.pone.0187267

Beckwith, L., Howard, J., Espinosa, M., & Tyler, R. (1999). Psychopathology, mother-child interaction, and infant development: Substance-abusing mothers and their offspring. *Special Issue: Developmental Approaches to Substance Use and Abuse*, *11*(4), 715–725. https://doi.org/http://dx.doi.org/10.1017/S095457949900228X

Bekkhus, M., Rutter, M., Barker, E. D., & Borge Mona; ORCID: http://orcid.org/0000-0002-4447-8909, A. I. H. A. I.-O. http://orcid. org/Bekkhu. (2011). The role of pre- and postnatal timing of family risk factors on child behavior at 36 months. *Journal of Abnormal Child Psychology*, *39*(4), 611–621. https://doi.org/http://dx.doi.org/10.1007/s10802-010-9477-z

Bennett, D. S., Bendersky, M., & Lewis, M. (2002). Children’s intellectual and emotional-behavioral adjustment at 4 years as a function of cocaine exposure, maternal characteristics, and environmental risk. *DEVELOPMENTAL PSYCHOLOGY*, *38*(5), 648–658. https://doi.org/10.1037//0012-1649.38.5.648

Betts, K. S., Williams, G. M., Najman, J. M., & Alati, R. (2014). Maternal depressive, anxious, and stress symptoms during pregnancy predict internalizing problems in adolescence. *Depression and Anxiety*, *31*(1), 9–18. https://doi.org/http://dx.doi.org/10.1002/da.22210

Betts, K. S., Williams, G. M., Najman, J. M., & Alati, R. (2015). The relationship between maternal depressive, anxious, and stress symptoms during pregnancy and adult offspring behavioral and emotional problems. *Depression and Anxiety*, *32*(2), 82–90. https://doi.org/http://dx.doi.org/10.1002/da.22272

Boyd, R. C., Zayas, L. H., & McKee, M. D. (2006). Mother-Infant Interaction, Life Events and Prenatal and Postpartum Depressive Symptoms Among Urban Minority Women in Primary Care. *Maternal and Child Health Journal*, *10*(2), 139–148. https://doi.org/http://dx.doi.org/10.1007/s10995-005-0042-2

Braungart-Rieker, J. M., Lefever, J. B., Planalp, E. M., & Moore Julia M.; ORCID: http://orcid.org/0000-0001-8027-3479, Planalp, Elizabeth M.; ORCID: http://orcid.org/0000-0001-9219-0806, E. S. A. I.-O. http://orcid. org/Braungar.-R. (2016). Body mass index at 3 years of age: Cascading effects of prenatal maternal depression and mother-infant dynamics. *The Journal of Pediatrics*, *177*, 128–132. https://doi.org/http://dx.doi.org/10.1016/j.jpeds.2016.06.023

Brennan, P. A., Hammen, C., Katz, A. R., & Le Brocque, R. M. (2002). Maternal depression, paternal psychopathology, and adolescent diagnostic outcomes. *Journal of Consulting and Clinical Psychology*, *70*(5), 1075–1085. https://doi.org/http://dx.doi.org/10.1037/0022-006X.70.5.1075

Brentani, A., & Fink, G. (2016). Maternal depression and child development: Evidence from Sao Paulo’s Western Region Cohort Study. *Revista Da Associacao Medica Brasileira (1992)*, *62*(6), 524–529. https://doi.org/https://dx.doi.org/10.1590/1806-9282.62.06.524

Burt, K. B., Hay, D. F., Pawlby, S., Harold, G., & Sharp, D. (2004). The prediction of disruptive behaviour disorders in an urban community sample: the contribution of person-centred analyses. *JOURNAL OF CHILD PSYCHOLOGY AND PSYCHIATRY*, *45*(6), 1159–1170. https://doi.org/10.1111/j.1469-7610.2004.00308.x

Burt, K. B., Van Dulmen, M. H. M., Carlivati, J., Egeland, B., Sroufe, L. A., Forman, D. R., … Carlson Manfred H. M.; ORCID: http://orcid.org/0000-0002-0018-4562, E. A. A. I.-O. http://orcid. org/Va. D. (2005). Mediating links between maternal depression and offspring psychopathology: The importance of independent data. *Journal of Child Psychology and Psychiatry*, *46*(5), 490–499. https://doi.org/http://dx.doi.org/10.1111/j.1469-7610.2004.00367.x

CAMPBELL, S. B., COHN, J. F., FLANAGAN, C., POPPER, S., & MEYERS, T. (1992). COURSE AND CORRELATES OF POSTPARTUM DEPRESSION DURING THE TRANSITION TO PARENTHOOD. *DEVELOPMENT AND PSYCHOPATHOLOGY*, *4*(1), 29–47. https://doi.org/10.1017/S095457940000554X

Campbell, S. B., Morgan-Lopez, A. A., Cox, M. J., & McLoyd, V. C. (2009). A latent class analysis of maternal depressive symptoms over 12 years and offspring adjustment in adolescence. *Journal of Abnormal Psychology*, *118*(3), 479–493. https://doi.org/http://dx.doi.org/10.1037/a0015923

Caplan, H. L., Cogill, S. R., Alexandra, H., Robson, K. M., Katz, R., & Kumar, R. (1989). Maternal depression and the emotional development of the child. *The British Journal of Psychiatry*, *154*, 818–822. https://doi.org/http://dx.doi.org/10.1192/bjp.154.6.818

Capron, L. E., Glover, V., Pearson, R. M., Evans, J., O’Connor, T. G., Stein, A., … Ramchandani Lauren E.; ORCID: http://orcid.org/0000-0002-7027-3077, Pearson, Rebecca M.; ORCID: http://orcid.org/0000-0001-8527-3400, Evans, Jonathan; ORCID: http://orcid.org/0000-0003-3171-640X, P. G. A. I.-O. http://orcid. org/Capro. (2015). Associations of maternal and paternal antenatal mood with offspring anxiety disorder at age 18 years. *Journal of Affective Disorders*, *187*, 20–26. https://doi.org/http://dx.doi.org/10.1016/j.jad.2015.08.012

Carter, A. S., Garrity-Rokous, F. E., Chazan-Cohen, R., Little, C., & Briggs-Gowan, M. J. (2001). Maternal depression and comorbidity: Predicting early parenting, attachment security, and toddler social-emotional problems and competencies. *Journal of the American Academy of Child & Adolescent Psychiatry*, *40*(1), 18–26. https://doi.org/http://dx.doi.org/10.1097/00004583-200101000-00012

Cents, R. A. M., Diamantopoulou, S., Hudziak, J. J., Jaddoe, V. W. V, Hofman, A., Verhulst, F. C., … H., T. (2013). Trajectories of maternal depressive symptoms predict child problem behaviour: The Generation R Study. *Psychological Medicine*, *43*(1), 13–25. https://doi.org/http://dx.doi.org/10.1017/S0033291712000657

Chiu, Y.-N., Gau, S. S.-F., Tsai, W.-C., Soong, W.-T., & Shang, C.-Y. (2009). Demographic and perinatal factors for behavioral problems among children aged 4-9 in Taiwan. *PSYCHIATRY AND CLINICAL NEUROSCIENCES*, *63*(4), 569–576. https://doi.org/10.1111/j.1440-1819.2009.01979.x

Choi, E. (2013). An exploratory study of maternal depressive symptoms and infant’s social skills in Korea: The mediating role of mothers’ social interactions with their infants. *Infant Mental Health Journal*, *34*(5), 458–469. https://doi.org/http://dx.doi.org/10.1002/imhj.21397

Chong, S.-C., Broekman, B. F. P., Qiu, A., Aris, I. M., Chan, Y. H., Rifkin-Graboi, A., … Chen, H. (2016). Anxiety and depression during pregnancy and temperament in early infancy: Findings from a multi-ethnic, Asian, prospective birth cohort study. *Infant Mental Health Journal*, *37*(5), 584–598. https://doi.org/http://dx.doi.org/10.1002/imhj.21582

Cogill, S. R., Caplan, H. L., Alexandra, H., Robson, K. M., & Kumar, R. (1986). Impact of maternal postnatal depression on cognitive development of young children. *British Medical Journal (Clinical Research Ed.)*, *292*(6529), 1165–1167. Retrieved from http://ovidsp.ovid.com/ovidweb.cgi?T=JS&PAGE=reference&D=med2&NEWS=N&AN=3085767

Davis, E. P., Glynn, L. M., Schetter, C. D., Hobel, C., Chicz-Demet, A., & Sandman, C. A. (2007). Prenatal exposure to maternal depression and cortisol influences infant temperament. *Journal of the American Academy of Child & Adolescent Psychiatry*, *46*(6), 737–746. https://doi.org/http://dx.doi.org/10.1097/chi.0b013e318047b775

Davis, E. P., Snidman, N., Wadhwa, P. D., Glynn, L. M., Schetter, C. D., & Sandman, C. A. (2004). Prenatal Maternal Anxiety and Depression Predict Negative Behavioral Reactivity in Infancy. *Infancy*, *6*(3), 319–331. https://doi.org/http://dx.doi.org/10.1207/s15327078in0603_1

de Almeida, C. P., Sa, E., Cunha, F., & Pires, E. (2012). Common mental disorders during pregnancy and baby’s development in the first year of life. *Journal of Reproductive and Infant Psychology*, *30*(4), 341–351. https://doi.org/http://dx.doi.org/10.1080/02646838.2012.736689

de Bruijn, A. T. C. E., van Bakel, H. J. A., & van Baar, A. L. (2009). Sex differences in the relation between prenatal maternal emotional complaints and child outcome. *EARLY HUMAN DEVELOPMENT*, *85*(5), 319–324. https://doi.org/10.1016/j.earlhumdev.2008.12.009

Deave, T., Heron, J., Evans, J., & Emond, A. (2008). The impact of maternal depression in pregnancy on early child development. *BJOG : An International Journal of Obstetrics and Gynaecology*, *115*(8), 1043–1051. https://doi.org/https://dx.doi.org/10.1111/j.1471-0528.2008.01752.x

der Waerden, J., Galera, C., Sutter-Dallay, A. L., Saurel-Cubizolles, M. J., Bernard, J. Y., De Agostini, M., … Melchior, M. (2015). Maternal depression and child development: evidence from an EDEN cohort. *EUROPEAN PSYCHIATRY*, *30*(8, S), S35. https://doi.org/10.1016/j.eurpsy.2015.09.101

der Waerden, J., & Melchior, M. (2014). MATERNAL DEPRESSION TIMING AND TRAJECTORIES, SOCIOECONOMIC POSITION AND CHILD PROBLEM BEHAVIOR AT AGE FIVE - RESULTS FROM THE EDEN MOTHER-CHILD COHORT. *EUROPEAN PSYCHIATRY*, *29*(1).

DiPietro, J. A., Novak, M. F. S. X., Costigan, K. A., Atella, L. D., & Reusing, S. P. (2006). Maternal Psychological Distress During Pregnancy in Relation to Child Development at Age Two. *Child Development*, *77*(3), 573–587. https://doi.org/http://dx.doi.org/10.1111/j.1467-8624.2006.00891.x

Eichler, A., Walz, L., Grunitz, J., Grimm, J., Van Doren, J., Raabe, E., … Moll Anna; ORCID: http://orcid.org/0000-0001-5584-0961, G. H. A. I.-O. http://orcid. org/Eichle. (2017). Children of prenatally depressed mothers: Externalizing and internalizing symptoms are accompanied by reductions in specific social-emotional competencies. *Journal of Child and Family Studies*, *26*(11), 3135–3144. https://doi.org/http://dx.doi.org/10.1007/s10826-017-0819-0

ENDENDIJK, J. J., Bruijn, A. T. C. E., Bakel, H. J. A., Wijnen, H. A. A., Pop, V. J. M., & Baar, A. L. (2017). Maternal interaction quality moderates effects of prenatal maternal emotional symptoms on girls’ internalizing problems. *Infant Mental Health Journal*, No-Specified. https://doi.org/http://dx.doi.org/10.1002/imhj.21662

Espinosa, M., Beckwith, L., Howard, J., Tyler, R., & Swanson, K. (2001). Maternal psychopathology and attachment in toddlers of heavy cocaine-using mothers. *INFANT MENTAL HEALTH JOURNAL*, *22*(3), 316–333. https://doi.org/10.1002/imhj.1004

Evans, J., Melotti, R., Heron, J., Ramchandani, P., Wiles, N., Murray, L., & Stein Jonathan; ORCID: http://orcid.org/0000-0003-3171-640X, Heron, Jon; ORCID: http://orcid.org/0000-0001-6199-5644, Ramchandani, Paul; ORCID: http://orcid.org/0000-0003-3646-2410, A. A. I.-O. http://orcid. org/Evan. (2012). The timing of maternal depressive symptoms and child cognitive development: A longitudinal study. *Journal of Child Psychology and Psychiatry*, *53*(6), 632–640. https://doi.org/http://dx.doi.org/10.1111/j.1469-7610.2011.02513.x

Fineberg, A. M. (2017). Maternal stress during pregnancy and adolescent depression: Spotlight on sex differences. *Dissertation Abstracts International: Section B: The Sciences and Engineering*, *78*(2–B(E)), No-Specified. Retrieved from http://ovidsp.ovid.com/ovidweb.cgi?T=JS&PAGE=reference&D=psyc13&NEWS=N&AN=2017-01061-043

Flach, C., Leese, M., Heron, J., Evans, J., Feder, G., Sharp, D., & Howard, L. M. (2011). Antenatal domestic violence, maternal mental health and subsequent child behaviour: a cohort study. *BJOG : An International Journal of Obstetrics and Gynaecology*, *118*(11), 1383–1391. https://doi.org/https://dx.doi.org/10.1111/j.1471-0528.2011.03040.x

Flouri, E., Ruddy, A., & Midouhas, E. (2017). Maternal depression and trajectories of child internalizing and externalizing problems: The roles of child decision making and working memory. *Psychological Medicine*, *47*(6), 1138–1148. https://doi.org/http://dx.doi.org/10.1017/S0033291716003226

Foster, C. E., Webster, M. C., Weissman, M. M., Pilowsky, D. J., Wickramaratne, P. J., Rush, A. J., … King Myrna M.; ORCID: http://orcid.org/0000-0003-3490-3075, Rush, A. John; ORCID: http://orcid.org/0000-0003-2004-2382, Alpert, Jonathan E.; ORCID: http://orcid.org/0000-0002-4332-908X, Wisniewski, Stephen R.; ORCID: http://orcid.org/0000-0002-3877-9860, C. A. A. I.-O. http://orcid. org/Weissma. (2008). Course and severity of maternal depression: Associations with family functioning and child adjustment. *Journal of Youth and Adolescence*, *37*(8), 906–916. https://doi.org/http://dx.doi.org/10.1007/s10964-007-9216-0

Foulon, S., Pingault, J.-B., Larroque, B., Melchior, M., Falissard, B., & Cote, S. M. (2015). Developmental predictors of inattention-hyperactivity from pregnancy to early childhood. *PloS One*, *10*(5), e0125996. https://doi.org/https://dx.doi.org/10.1371/journal.pone.0125996

Gajos, J. M., & Beaver, K. M. (2017). Maternal depression and risk for antisocial behaviour in children. *Child & Family Social Work*, *22*(1), 349–363. https://doi.org/http://dx.doi.org/10.1111/cfs.12247

Gerardin, P. (2012). Characteristics and clinical consequences of prenatal depression. Main results of a prospective case-control study on perinatal depression from pregnancy to one year-old infant. *Specificite et Enjeux de La Depression de La Grossesse. Principaux Resultats D’une Recherche Longitudinale Sur Les Depressions Du Peripartum, Du Troisieme Trimestre de Grossesse Au 12e Mois de L’enfant.*, *60*(2), 138–146. https://doi.org/http://dx.doi.org/10.1016/j.neurenf.2011.10.006

Gerardin, P., Wendland, J., Bodeau, N., Galin, A., Bialobos, S., Tordjman, S., … Cohen Jacky; ORCID: http://orcid.org/0000-0003-1537-960X, D. A. I.-O. http://orcid. org/Nizar. (2011). Depression during pregnancy: Is the developmental impact earlier in boys? A prospective case-control study. *The Journal of Clinical Psychiatry*, *72*(3), 378–387. https://doi.org/http://dx.doi.org/10.4088/JCP.09m05724blu

Giallo, R., Woolhouse, H., Gartland, D., Hiscock, H., & Brown Harriet; ORCID: http://orcid.org/0000-0003-3017-2770, S. A. I.-O. http://orcid. org/Hiscoc. (2015). The emotional-behavioural functioning of children exposed to maternal depressive symptoms across pregnancy and early childhood: A prospective Australian pregnancy cohort study. *European Child & Adolescent Psychiatry*, *24*(10), 1233–1244. https://doi.org/http://dx.doi.org/10.1007/s00787-014-0672-2

Gjerde, L. C., Eilertsen, E., McAdams, T., Rijsdijk, F., & Ystrom, E. (2017). Disentangling the effects of maternal depressive symptoms to child internalizing and externalizing problems: an extended children of twins study. *BEHAVIOR GENETICS*, *47*(6), 697.

Glasheen, C., Richardson, G. A., Kim, K. H., Larkby, C. A., Swartz, H. A., & Day Holly A.; ORCID: http://orcid.org/0000-0002-7611-9900, N. L. A. I.-O. http://orcid. org/Swart. (2013). Exposure to maternal pre- and postnatal depression and anxiety symptoms: Risk for major depression, anxiety disorders, and conduct disorder in adolescent offspring. *Development and Psychopathology*, *25*(4), 1045–1063. https://doi.org/http://dx.doi.org/10.1017/S0954579413000369

Guyon-Harris, K., Huth-Bocks, A., Lauterbach, D., & Janisse, H. (2016). Trajectories of maternal depressive symptoms across the birth of a child: Associations with toddler emotional development. *Archives of Women’s Mental Health*, *19*(1), 153–165. https://doi.org/http://dx.doi.org/10.1007/s00737-015-0546-8

Hammen, C., & Brennan, P. A. (2003). Severity, chronicity, and timing of maternal depression and risk for adolescent offspring diagnoses in a community sample. *Archives of General Psychiatry*, *60*(3), 253–258. Retrieved from http://ovidsp.ovid.com/ovidweb.cgi?T=JS&PAGE=reference&D=med4&NEWS=N&AN=12622658

Hammerton, G., Mahedy, L., Mars, B., Harold, G. T., Thapar, A., Zammit, S., & Collishaw, S. (2015). Association between Maternal Depression Symptoms across the First Eleven Years of Their Child’s Life and Subsequent Offspring Suicidal Ideation. *PLOS ONE*, *10*(7). https://doi.org/10.1371/journal.pone.0131885

Hammerton, G., Zammit, S., Mahedy, L., Pearson, R. M., Sellers, R., Thapar, A., & Collishaw, S. (2015). Pathways to Suicide-Related Behavior in Offspring of Mothers With Depression: The Role of Offspring Psychopathology. *JOURNAL OF THE AMERICAN ACADEMY OF CHILD AND ADOLESCENT PSYCHIATRY*, *54*(5), 385–393. https://doi.org/10.1016/j.jaac.2015.02.006

Hay, D. F., & Pawlby, S. (2003). Prosocial development in relation to children’s and mothers’ psychological problems. *Child Development*, *74*(5), 1314–1327. https://doi.org/http://dx.doi.org/10.1111/1467-8624.00609

Hayes, L. J., Goodman, S. H., & Carlson, E. (2013). Maternal antenatal depression and infant disorganized attachment at 12 months. *Attachment & Human Development*, *15*(2), 133–153. https://doi.org/http://dx.doi.org/10.1080/14616734.2013.743256

Heron, J., Barker, E. D., Joinson, C., Lewis, G., Hickman, M., Munafo, M., & Macleod Jon; ORCID: http://orcid.org/0000-0001-6199-5644, Lewis, Glyn; ORCID: http://orcid.org/0000-0001-5205-8245, Munafo, Marcus; ORCID: http://orcid.org/0000-0002-4049-993X, J. A. I.-O. http://orcid. org/Hero. (2013). Childhood conduct disorder trajectories, prior risk factors and cannabis use at age 16: Birth cohort study. *Addiction*, *108*(12), 2129–2138. https://doi.org/http://dx.doi.org/10.1111/add.12268

Hipwell, A. E., Murray, J., Xiong, S., Stepp, S. D., & Keenan, K. E. (2016). Effects of Adolescent Childbearing on Maternal Depression and Problem Behaviors: A Prospective, Population-Based Study Using Risk-Set Propensity Scores. *PloS One*, *11*(5), e0155641. https://doi.org/https://dx.doi.org/10.1371/journal.pone.0155641

Huot, R. L., Brennan, P. A., Stowe, Z. N., Plotsky, P. M., & Walker, E. F. (2004). Negative Affect in Offspring of Depressed Mothers Is Predicted by Infant Cortisol Levels at 6 Months and Maternal Depression during Pregnancy, but Not Postpartum. *Biobehavioral Stress Response: Protective and Damaging Effects.*, 234–236. Retrieved from http://ovidsp.ovid.com/ovidweb.cgi?T=JS&PAGE=reference&D=psyc4&NEWS=N&AN=2005-01907-030

Junge, C., Garthus-Niegel, S., Slinning, K., Polte, C., Simonsen, T. B., & Eberhard-Gran, M. (2017). The impact of perinatal depression on children’s social-emotional development: A longitudinal study. *Maternal and Child Health Journal*, *21*(3), 607–615. https://doi.org/http://dx.doi.org/10.1007/s10995-016-2146-2

Kahn, R. S., Wilson, K. X., & Wise, P. H. (2005). Intergenerational health disparities: Socioeconomic status, women’s health conditions, and child behavior problems. *PUBLIC HEALTH REPORTS*, *120*(4), 399–408. https://doi.org/10.1177/003335490512000407

Kaplan, L. A., Evans, L., & Monk, C. (2008). Effects of mothers’ prenatal psychiatric status and postnatal caregiving on infant biobehavioral regulation: Can prenatal programming be modified? *Early Human Development*, *84*(4), 249–256. https://doi.org/http://dx.doi.org/10.1016/j.earlhumdev.2007.06.004

Karam, F., Sheehy, O., Huneau, M.-C., Chambers, C., Fraser, W. D., Johnson, D., … Berard, A. (2016). Impact of maternal prenatal and parental postnatal stress on 1-year-old child development: Results from the OTIS antidepressants in pregnancy study. *Archives of Women’s Mental Health*, *19*(5), 835–843. https://doi.org/http://dx.doi.org/10.1007/s00737-016-0624-6

Kemppinen, K., Kumpulainen, K., Raita-Hasu, J., Moilanen, I., & Ebeling, H. (2006). The continuity of maternal sensitivity from infancy to toddler age. *Journal of Reproductive and Infant Psychology*, *24*(3), 199–212. https://doi.org/http://dx.doi.org/10.1080/02646830600821249

Kiernan, K. E., & Huerta, M. C. (2008). Economic deprivation, maternal depression, parenting and children’s cognitive and emotional development in early childhood. *British Journal of Sociology*, *59*(4), 783–806. https://doi.org/http://dx.doi.org/10.1111/j.1468-4446.2008.00219.x

Kiernan, K. E., & Mensah, F. K. (2009). Poverty, Maternal Depression, Family Status and Children’s Cognitive and Behavioural Development in Early Childhood: A Longitudinal Study. *JOURNAL OF SOCIAL POLICY*, *38*, 569–588. https://doi.org/10.1017/S0047279409003250

Kim-Cohen, J., Moffitt, T. E., Taylor, A., Pawlby, S. J., & Caspi Terrie E.; ORCID: http://orcid.org/0000-0002-8589-6760, A. A. I.-O. http://orcid. org/Moffit. (2005). Maternal Depression and Children’s Antisocial Behavior: Nature and Nurture Effects. *Archives of General Psychiatry*, *62*(2), 173–181. https://doi.org/http://dx.doi.org/10.1001/archpsyc.62.2.173

Korhonen, M., Luoma, I., Salmelin, R., Helminen, M., Nummi, T., & Tamminen, T. (2013). Trajectories of maternal depressive symptoms and adolescent emotional and behavioural problems. *EUROPEAN CHILD & ADOLESCENT PSYCHIATRY*, *22*(2), S250.

Korhonen, M., Luoma, I., Salmelin, R., & Tamminen, T. (2013). Maternal antenatal expectations and postnatal perceptions of the baby and adolescent internalising and externalising problems. *EUROPEAN CHILD & ADOLESCENT PSYCHIATRY*, *22*(2), S253–S254.

Koutra, K., Chatzi, L., Bagkeris, M., Vassilaki, M., Bitsios, P., & Kogevinas, M. (2013). Antenatal and postnatal maternal mental health as determinants of infant neurodevelopment at 18 months of age in a mother-child cohort (Rhea Study) in Crete, Greece. *Social Psychiatry and Psychiatric Epidemiology*, *48*(8), 1335–1345. https://doi.org/http://dx.doi.org/10.1007/s00127-012-0636-0

Koutra, K., Roumeliotaki, T., Kyriklaki, A., Kampouri, M., Sarri, K., Vassilaki, M., … Chatzi, L. (2017). Maternal depression and personality traits in association with child neuropsychological and behavioral development in preschool years: Mother-child cohort (Rhea Study) in Crete, Greece. *Journal of Affective Disorders*, *217*, 89–98. https://doi.org/http://dx.doi.org/10.1016/j.jad.2017.04.002

Kvalevaag, A. L., Ramchandani, P. G., Hove, O., Eberhard-Gran, M., Assmus, J., Havik, O. E., … Biringer, E. (2015). Parents’ Prenatal Mental Health and Emotional, Behavioral and Social Development in Their Children. *CHILD PSYCHIATRY & HUMAN DEVELOPMENT*, *46*(6), 874–883. https://doi.org/10.1007/s10578-014-0527-6

Lanzi, R. G., Bert, S. C., & Jacobs, B. K. (2009). Depression among a sample of first-time adolescent and adult mothers. *Journal of Child and Adolescent Psychiatric Nursing*, *22*(4), 194–202. https://doi.org/http://dx.doi.org/10.1111/j.1744-6171.2009.00199.x

LAUCHT, M., ESSER, G., & SCHMIDT, M. H. (1994). PARENTAL MENTAL DISORDER AND EARLY CHILD-DEVELOPMENT. *EUROPEAN CHILD & ADOLESCENT PSYCHIATRY*, *3*(3), 125–137. https://doi.org/10.1007/BF02720321

Lee, L. C., Newschaffer, C. J., Halpern, C. T., & Hertz-Picciotto, I. (2005). Effect of maternal depressive symptoms on their ratings of toddlers’ behavior problems. *AMERICAN JOURNAL OF EPIDEMIOLOGY*, *161*(11, S), S143.

Leis, J. A. (2012). Perinatal mental health problems: Maternal and child outcomes and implications for prevention. *Dissertation Abstracts International: Section B: The Sciences and Engineering*, *72*(10–B), 5877. Retrieved from http://ovidsp.ovid.com/ovidweb.cgi?T=JS&PAGE=reference&D=psyc9&NEWS=N&AN=2012-99080-101

Letourneau, N. L., Tramonte, L., & Willms, J. D. (2013). Maternal depression, family functioning and children’s longitudinal development. *Journal of Pediatric Nursing*, *28*(3), 223–234. https://doi.org/https://dx.doi.org/10.1016/j.pedn.2012.07.014

Lin, Y., Xu, J., Huang, J., Jia, Y., Zhang, J., Yan, C., & Zhang, J. (2017). Effects of prenatal and postnatal maternal emotional stress on toddlers’ cognitive and temperamental development. *Journal of Affective Disorders*, *207*, 9–17. https://doi.org/http://dx.doi.org/10.1016/j.jad.2016.09.010

Loeber, R., Hipwell, A., Battista, D., Sembower, M., & Stouthamer-Loeber, M. (2009). Intergenerational Transmission of Multiple Problem Behaviors: Prospective Relationships between Mothers and Daughters. *JOURNAL OF ABNORMAL CHILD PSYCHOLOGY*, *37*(8), 1035–1048. https://doi.org/10.1007/s10802-009-9337-x

Lusby, C. M., Goodman, S. H., Yeung, E. W., Bell, M. A., & Stowe, Z. N. (2016). Infant EEG and temperament negative affectivity: Coherence of vulnerabilities to mothers’ perinatal depression. *Development and Psychopathology*, *28*(4), 895–911. https://doi.org/http://dx.doi.org/10.1017/S0954579416000614

Mars, B., Collishaw, S., Hammerton, G., Rice, F., Harold, G. T., Smith, D., … Thapar, A. (2015). Longitudinal symptom course in adults with recurrent depression: Impact on impairment and risk of psychopathology in offspring. *JOURNAL OF AFFECTIVE DISORDERS*, *182*, 32–38. https://doi.org/10.1016/j.jad.2015.04.018

Matijasevich, A., Murray, J., Cooper, P. J., Anselmi, L., Barros, A. J. D., Barros, F. C., & Santos, I. S. (2015). Trajectories of maternal depression and offspring psychopathology at 6 years: 2004 Pelotas cohort study. *Journal of Affective Disorders*, *174*, 424–431. https://doi.org/10.1016/j.jad.2014.12.012

McDonnell, C. G., & Valentino, K. (2016). Intergenerational effects of childhood trauma: Evaluating pathways among maternal ACEs, perinatal depressive symptoms, and infant outcomes. *Child Maltreatment*, *21*(4), 317–326. https://doi.org/http://dx.doi.org/10.1177/1077559516659556

McGrath, J. M., Records, K., & Rice, M. (2008). Maternal depression and infant temperament charactenstics. *INFANT BEHAVIOR & DEVELOPMENT*, *31*(1), 71–80. https://doi.org/10.1016/j.infbeh.2007.07.001

Meehan, A. J., Maughan, B., Cecil, C. A. M., & Barker, E. D. (2017). Interpersonal callousness and co-occurring anxiety: Developmental validity of an adolescent taxonomy. *Journal of Abnormal Psychology*, *126*(2), 225–236. https://doi.org/http://dx.doi.org/10.1037/abn0000235

Melchior, M., Chastang, J.-F., de Lauzon, B., Galera, C., Saurel-Cubizolles, M.-J., & Larroque, B. (2012). Maternal depression, socioeconomic position, and temperament in early childhood: The EDEN mother-child cohort. *Journal of Affective Disorders*, *137*(1–3), 165–169. https://doi.org/http://dx.doi.org/10.1016/j.jad.2011.09.018

Mohan, D. (2013). The relationship between maternal depression (ante natal and pre-school stage) and childhood behavioural problems. *EUROPEAN CHILD & ADOLESCENT PSYCHIATRY*, *22*(2), S98–S99.

Mohan, D., Fitzgerald, M., & Collins, C. (1998). The relationship between maternal depression (antenatal and pre-school stage) and childhood behavioural problems. *Irish Journal of Psychological Medicine*, *15*(1), 10–13. https://doi.org/http://dx.doi.org/10.1017/S0790966700004602

Muzik, M. (2010). MATERNAL PERINATAL DEPRESSION: IMPACT ON INFANT EMOTION REGULATION AND LATER TODDLER BEHAVIOR PROBLEMS. *EUROPEAN PSYCHIATRY*, *25*(1).

NAJMAN, J. M., WILLIAMS, G. M., NIKLES, J., SPENCE, S., BOR, W., O’CALLAGHAN, M., … ANDERSEN, M. J. (2000). Mothers’ Mental Illness and Child Behavior Problems: Cause-Effect Association or Observation Bias? *Journal of the American Academy of Child & Adolescent Psychiatry*, *39*(5), 592–602. https://doi.org/10.1097/00004583-200005000-00013

Nolvi, S., Karlsson, L., Bridgett, D. J., Korja, R., Huizink, A. C., Kataja, E.-L., & Karlsson, H. (2016). Maternal prenatal stress and infant emotional reactivity six months postpartum. *Journal of Affective Disorders*, *199*, 163–170. https://doi.org/http://dx.doi.org/10.1016/j.jad.2016.04.020

Norcross, P. L., Leerkes, E. M., & Zhou, N. (2017). Examining pathways linking maternal depressive symptoms in infancy to children’s behavior problems: The role of maternal unresponsiveness and negative behaviors. *Infant Behavior & Development*, *49*, 238–247. https://doi.org/http://dx.doi.org/10.1016/j.infbeh.2017.09.009

O’Connor, E. E., Langer, D. A., & Tompson, M. C. (2017). Maternal depression and youth internalizing and externalizing symptomatology: Severity and chronicity of past maternal depression and current maternal depressive symptoms. *Journal of Abnormal Child Psychology*, *45*(3), 557–568. https://doi.org/http://dx.doi.org/10.1007/s10802-016-0185-1

O’Connor, T. G., Heron, J., Golding, J., Beveridge, M., & Glover, V. (2002). Maternal antenatal anxiety and children’s behavioural/emotional problems at 4 years: Report from the Avon Longitudinal Study of Parents and Children. *The British Journal of Psychiatry*, *180*(6), 502–508. https://doi.org/http://dx.doi.org/10.1192/bjp.180.6.502

O’Connor, T. G., Heron, J., Golding, J., & Glover, V. (2003). Maternal antenatal anxiety and behavioural/emotional problems in children: A test of a programming hypothesis. *Journal of Child Psychology and Psychiatry*, *44*(7), 1025–1036. https://doi.org/http://dx.doi.org/10.1111/1469-7610.00187

O’Leary, N., Jairaj, C., Farrell, C., Doolin, K., & O’Keane, V. (2016). Prenatal depression: the relationship between maternal gestational cortisol and infant neurobehavioural development at 2 months. *EUROPEAN NEUROPSYCHOPHARMACOLOGY*, *26*(2), S192. https://doi.org/10.1016/S0924-977X(16)31030-6

Otake, Y., Nakajima, S., Uno, A., Kato, S., Sasaki, S., Yoshioka, E., … Kishi, R. (2014). Association between maternal antenatal depression and infant development: a hospital-based prospective cohort study. *Environmental Health and Preventive Medicine*, *19*(1), 30–45. https://doi.org/https://dx.doi.org/10.1007/s12199-013-0353-7

Park, S., Kim, B.-N., Kim, J.-W., Shin, M.-S., Yoo, H. J., Lee, J., & Cho, S.-C. (2014). Associations between maternal stress during pregnancy and offspring internalizing and externalizing problems in childhood. *International Journal of Mental Health Systems*, *8*. Retrieved from http://ovidsp.ovid.com/ovidweb.cgi?T=JS&PAGE=reference&D=psyc11&NEWS=N&AN=2015-19531-001

Pawlby, S., Hay, D., Sharp, D., Waters, C. S., & Pariante, C. M. (2011). Antenatal depression and offspring psychopathology: The influence of childhood maltreatment. *The British Journal of Psychiatry*, *199*(2), 106–112. https://doi.org/http://dx.doi.org/10.1192/bjp.bp.110.087734

Perry, M. A., & Fantuzzo, J. W. (2010). A multivariate investigation of maternal risks and their relationship to low-income, preschool children’s competencies. *Applied Developmental Science*, *14*(1), 1–17. https://doi.org/http://dx.doi.org/10.1080/10888690903510281

Pihlakoski, L., Sourander, A., Aromaa, M., Ronning, J. A., Rautava, P., Helenius, H., & Sillanpaa Andre; ORCID: http://orcid.org/0000-0003-0361-7244, M. A. I.-O. http://orcid. org/Sourande. (2013). Do antenatal and postnatal parental psychological distress, and recognized need of help predict preadolescent’s psychiatric symptoms? The Finnish Family Competence Cohort Study. *Child Psychiatry and Human Development*, *44*(2), 305–319. https://doi.org/http://dx.doi.org/10.1007/s10578-012-0326-x

Pina-Camacho, L., Jensen, S. K., Gaysina, D., & Barker L.; ORCID: http://orcid.org/0000-0003-1960-6443, Gaysina, D.; ORCID: http://orcid.org/0000-0002-9283-5667, E. D. A. I.-O. http://orcid. org/Pin.-C. (2015). Maternal depression symptoms, unhealthy diet and child emotional-behavioural dysregulation. *Psychological Medicine*, *45*(9), 1851–1860. https://doi.org/http://dx.doi.org/10.1017/S0033291714002955

Plant, D. T., Barker, E. D., Waters, C. S., Pawlby, S., & Pariante D. T.; ORCID: http://orcid.org/0000-0003-0791-158X, C. M. A. I.-O. http://orcid. org/Plan. (2013). Intergenerational transmission of maltreatment and psychopathology: The role of antenatal depression. *Psychological Medicine*, *43*(3), 519–528. Retrieved from http://ovidsp.ovid.com/ovidweb.cgi?T=JS&PAGE=reference&D=psyc10&NEWS=N&AN=2013-04255-006

Plant, D. T., Jones, F. W., Pariante, C. M., & Pawlby, S. (2017). Association between maternal childhood trauma and offspring childhood psychopathology: mediation analysis from the ALSPAC cohort. *The British Journal of Psychiatry : The Journal of Mental Science*, *211*(3), 144–150. https://doi.org/https://dx.doi.org/10.1192/bjp.bp.117.198721

Prady, S. L., Pickett, K. E., Croudace, T., Mason, D., Petherick, E. S., McEachan, R. R. C., … Wright, J. (2016). Maternal psychological distress in primary care and association with child behavioural outcomes at age three. *EUROPEAN CHILD & ADOLESCENT PSYCHIATRY*, *25*(6), 601–613. https://doi.org/10.1007/s00787-015-0777-2

Ramchandani, P. G., Richter, L. M., Norris, S. A., & Stein Paul G.; ORCID: http://orcid.org/0000-0003-3646-2410, A. A. I.-O. http://orcid. org/Ramchandan. (2010). Maternal prenatal stress and later child behavioral problems in an urban South African setting. *Journal of the American Academy of Child & Adolescent Psychiatry*, *49*(3), 239–247. https://doi.org/http://dx.doi.org/10.1097/00004583-201003000-00007

Robinson, M., Oddy, W. H., Li, J., Kendall, G. E., de Klerk, N. H., Silburn, S. R., … Mattes, E. (2008). Pre- and postnatal influences on preschool mental health: a large-scale cohort study. *Journal of Child Psychology and Psychiatry, and Allied Disciplines*, *49*(10), 1118–1128. https://doi.org/https://dx.doi.org/10.1111/j.1469-7610.2008.01955.x

Rode, J. L., & Kiel Jennifer L.; ORCID: http://orcid.org/0000-0003-2707-0538, E. J. A. I.-O. http://orcid. org/Rod. (2016). The mediated effects of maternal depression and infant temperament on maternal role. *Archives of Women’s Mental Health*, *19*(1), 133–140. https://doi.org/http://dx.doi.org/10.1007/s00737-015-0540-1

Rouse, M. H., & Goodman, S. H. (2014). Perinatal depression influences on infant negative affectivity: Timing, severity, and co-morbid anxiety. *Infant Behavior & Development*, *37*(4), 739–751. https://doi.org/http://dx.doi.org/10.1016/j.infbeh.2014.09.001

Santos Jr., H., Yang, Q., Docherty, S. L., White-Traut, R., & Holditch-Davis, D. (2016). Relationship of Maternal Psychological Distress Classes to Later Mother-Infant Interaction, Home Environment, and Infant Development in Preterm Infants. *RESEARCH IN NURSING & HEALTH*, *39*(3), 175–186. https://doi.org/10.1002/nur.21719

Santos, I. S., Matijasevich, A., Barros, A. J. D., & Barros Aluisio J. D.; ORCID: http://orcid.org/0000-0002-2022-8729, F. C. A. I.-O. http://orcid. org/Barro. (2014). Antenatal and postnatal maternal mood symptoms and psychiatric disorders in pre-school children from the 2004 Pelotas Birth Cohort. *Journal of Affective Disorders*, *164*, 112–117. https://doi.org/http://dx.doi.org/10.1016/j.jad.2014.04.033

Servili, C., Medhin, G., Hanlon, C., Tomlinson, M., Worku, B., Baheretibeb, Y., … Prince, M. (2010). Maternal common mental disorders and infant development in Ethiopia: the P-MaMiE Birth Cohort. *BMC PUBLIC HEALTH*, *10*. https://doi.org/10.1186/1471-2458-10-693

Shakel, N., & Shakel, N. (2017). Prenatal exposure to maternal depression and its influences on infant development and behaviour. *EUROPEAN PSYCHIATRY*, *41*(S), S363–S364. https://doi.org/10.1016/j.eurpsy.2017.02.490

Sirvinskiene, G., Zemaitiene, N., Jusiene, R., & Markuniene, E. (2016). Predictors of emotional and behavioral problems in 1-year-old children: A longitudinal perspective. *Infant Mental Health Journal*, *37*(4), 401–410. https://doi.org/http://dx.doi.org/10.1002/imhj.21575

Stroustrup, A., Hsu, H.-H., Svensson, K., Schnaas, L., Cantoral, A., Solano Gonzalez, M., … Wright, R. J. (2016). Toddler temperament and prenatal exposure to lead and maternal depression. *Environmental Health : A Global Access Science Source*, *15*(1), 71. https://doi.org/https://dx.doi.org/10.1186/s12940-016-0147-7

Sullivan, E. L., Holton, K. F., Nousen, E. K., Barling, A. N., Sullivan, C. A., Propper, C. B., & Nigg, J. T. (2015). Early identification of ADHD risk via infant temperament and emotion regulation: A pilot study. *Journal of Child Psychology and Psychiatry*, *56*(9), 949–957. https://doi.org/http://dx.doi.org/10.1111/jcpp.12426

Szekely, E., Tiemeier, H., Jansen, P. W., Jaddoe, V. W. V, Hofman, A., Verhulst, F. C., & Herba, C. M. (2014). Maternal Depressive Symptoms Are Associated With Low Fearfulness in Preschoolers. *JOURNAL OF CLINICAL CHILD AND ADOLESCENT PSYCHOLOGY*, *43*(5), 791–798. https://doi.org/10.1080/15374416.2013.862800

Tao, H., Shao, T., Ni, L., Sun, Y., Yan, S., Gu, C., … Tong, S. (2016). [The relationship between maternal emotional symptoms during pregnancy and emotional and behavioral problems in preschool children: a birth cohort study]. *Zhonghua Yu Fang Yi Xue Za Zhi [Chinese Journal of Preventive Medicine]*, *50*(2), 129–135. https://doi.org/https://dx.doi.org/10.3760/cma.j.issn.0253-9624.2016.02.006

Tharner, A., Luijk, M. P. C. M., van IJzendoorn, M. H., Bakermans-Kranenburg, M. J., Jaddoe, V. W. V, Hofman, A., … Tiemeier Henning; ORCID: http://orcid.org/0000-0002-4395-1397, H. A. I.-O. http://orcid. org/Tiemeie. (2012). Maternal lifetime history of depression and depressive symptoms in the prenatal and early postnatal period do not predict infant-mother attachment quality in a large, population-based Dutch cohort study. *Attachment & Human Development*, *14*(1), 63–81. https://doi.org/http://dx.doi.org/10.1080/14616734.2012.636659

Tran, T. D., Biggs, B.-A., Tran, T., Simpson, J. A., de Mello, M. C., Hanieh, S., … Fisher Thach Duc; ORCID: http://orcid.org/0000-0002-4686-8601, Biggs, Beverley-Ann; ORCID: http://orcid.org/0000-0002-2961-9793, Simpson, Julie Anne; ORCID: http://orcid.org/0000-0002-2660-2013, Fisher, Jane; ORCID: http://orcid.org/0000-0002-1959-6807, J. A. I.-O. http://orcid. org/Tra. (2014). Perinatal common mental disorders among women and the social and emotional development of their infants in rural Vietnam. *Journal of Affective Disorders*, *160*, 104–112. https://doi.org/http://dx.doi.org/10.1016/j.jad.2013.12.034

Turney, K. (2011). Chronic and proximate depression among mothers: Implications for child well-being. *Journal of Marriage and Family*, *73*(1), 149–163. https://doi.org/http://dx.doi.org/10.1111/j.1741-3737.2010.00795.x

Turney, K. (2012). Pathways of disadvantage: Explaining the relationship between maternal depression and children’s problem behaviors. *Social Science Research*, *41*(6), 1546–1564. https://doi.org/http://dx.doi.org/10.1016/j.ssresearch.2012.06.003

Vanska, M., Punamaki, R.-L., Lindblom, J., Flykt, M., Tolvanen, A., Unkila-Kallio, L., … Tiitinen, A. (2017). Parental pre- and postpartum mental health predicts child mental health and development. *Family Relations: An Interdisciplinary Journal of Applied Family Studies*, No-Specified. https://doi.org/http://dx.doi.org/10.1111/fare.12260

Vanska, M., Punamaki, R.-L., Tolvanen, A., Lindblom, J., Flykt, M., Unkila-Kallio, L., … Tulppala, M. (2011). Maternal pre- and postnatal mental health trajectories and child mental health and development: Prospective study in a normative and formerly infertile sample. *INTERNATIONAL JOURNAL OF BEHAVIORAL DEVELOPMENT*, *35*(6), 517–531. https://doi.org/10.1177/0165025411417505

Velders, F. P., Dieleman, G., Henrichs, J., Jaddoe, V. W. V, Hofman, A., Verhulst, F. C., … Tiemeier James J.; ORCID: http://orcid.org/0000-0001-9653-758X, Tiemeier, Henning; ORCID: http://orcid.org/0000-0002-4395-1397, H. A. I.-O. http://orcid. org/Hudzia. (2011). Prenatal and postnatal psychological symptoms of parents and family functioning: The impact on child emotional and behavioural problems. *European Child & Adolescent Psychiatry*, *20*(7), 341–350. https://doi.org/http://dx.doi.org/10.1007/s00787-011-0178-0

Werner, E. A. (2009). The effects of pregnant women’s mood, stress and cortisol levels on infant reactivity: A test of the fetal programming hypothesis. *Dissertation Abstracts International: Section B: The Sciences and Engineering*, *69*(10–B), 6441. Retrieved from http://ovidsp.ovid.com/ovidweb.cgi?T=JS&PAGE=reference&D=psyc6&NEWS=N&AN=2009-99080-128

Wilkins, A. J., O’Callaghan, M. J., Najman, J. M., Bor, W., Williams, G. M., & Shuttlewood, G. (2004). Early childhood factors influencing health-related quality of life in adolescents at 13 years. *Journal of Paediatrics and Child Health*, *40*(3), 102–109. <https://doi.org/http://dx.doi.org/10.1111/j.1440-1754.2004.00309.x>

Wolford, E., Lahti, M., Tuovinen, S., Lahti, J., Lipsanen, J., Savolainen, K., … Raikkonen, K. (2017). Maternal depressive symptoms during and after pregnancy are associated with attention-deficit/hyperactivity disorder symptoms in their 3- to 6-year-old children. *PloS One*, *12*(12), e0190248. https://doi.org/https://dx.doi.org/10.1371/journal.pone.0190248

Wu, P.-C., Kim, H., Viner-Brown, S., & High, P. (2013). Is Maternal Depression Associated with Increased Behavioral/Developmental Problems in Toddlers? *JOURNAL OF DEVELOPMENTAL AND BEHAVIORAL PEDIATRICS*, *34*(6), S9.

Zeiders, K. H., Umana-Taylor, A. J., Jahromi, L. B., Updegraff, K. A., & White, R. M. B. (2016). Discrimination and acculturation stress: A longitudinal study of children’s well-being from prenatal development to 5 years of age. *Journal of Developmental and Behavioral Pediatrics*, *37*(7), 557–564. https://doi.org/http://dx.doi.org/10.1097/DBP.0000000000000321
